# Beyond the Socioeconomic Gradient: The Epigenetic Coefficient (EGC) as a Distribution-Sensitive Framework for Measuring Inequality in Epigenetic Ageing

**DOI:** 10.3390/life16101720

**Published:** 2026-10-15

**Authors:** Piergiorgio Marigliano, Mario Capunzo, Silvana Mirella Aliberti

**Affiliations:** 1 Department of Economics, ECampus University, 22060 Novedrate, Italy; piergiorgio.marigliano@uniecampus.it; 2 Department of Medicine, Surgery and Dentistry “Scuola Medica Salernitana”, University of Salerno, 84081 Baronissi, Italy; mcapunzo@unisa.it; 3 Complex Operational Unit of Health and Hygiene, University Hospital “San Giovanni di Dio e Ruggi d’Aragona”, 84131 Salerno, Italy; 4 Division of Preventive Medicine, Dresden International University (DIU), 01067 Dresden, Germany

**Keywords:** epigenetic age acceleration, DNA methylation clocks, socioeconomic inequality, biological ageing, Wasserstein distance, health equity, SDG 3, SDG 10

## Abstract

Background/Objectives: Studies of socioeconomic differences in DNA methylation-based ageing mainly report regression coefficients, group contrasts, mediation estimates, or decompositions, which do not describe how complete epigenetic age acceleration (EAA) distributions differ across ordered socioeconomic strata. We developed the Epigenetic Coefficient (EGC) to address this gap. Methods: The study comprised two linked workstreams. First, we specified the EGC profile (EGC-M, a distributional magnitude based on weighted pairwise Wasserstein-1 distances; EGC-O, a conditional orientation; EGC-S, a rank-gradient companion; and L, the population mean burden) and verified the frozen version 1.0 point estimators through independent R (version 4.6.1) and Python (version 3.14.5) implementations, controlled datasets, and a small exploratory pilot. Second, a structured evidence map of 238 deduplicated publications was searched for equivalent methods. Results: R and Python agreed in 1235/1235 prespecified comparisons across 20 fixed datasets (maximum absolute difference 3.29 × 10^−14^). EGC-M detected distributional differences between strata with equal means. The pilot (*N* = 2500; five replications per condition) behaved coherently under null and gradient conditions but was not designed to estimate operating characteristics. In the evidence map, none of the 160 relevant publications described an equivalent coefficient, and eight near-direct precedents were audited in full text. Conclusions: The EGC is mathematically specified and reproducible across software. Its inferential calibration, biological validity, and incremental value over established inequality measures remain to be tested; it is therefore a candidate research framework.

## 1. Introduction

Population ageing and the unequal distribution of healthy life expectancy are joint public-health challenges. The 2030 Agenda places health and well-being under SDG 3 and the reduction in inequalities under SDG 10, but existing monitoring systems primarily rely on demographic, clinical, behavioral, economic, and service indicators [1,2,3]. These measures are indispensable, yet they may not capture the biological embodiment of cumulative social conditions before overt disease becomes clinically visible.

DNA methylation-based epigenetic clocks estimate chronological age, phenotypic age, mortality-related ageing, or the pace of ageing from methylation patterns [4,5,6,7,8]. Epigenetic age acceleration (EAA) is commonly represented as the deviation or residual of DNA methylation age from chronological age, although its exact definition depends on the clock and analytic protocol. Because clock generations capture different biological and technical features, EAA is not a single interchangeable outcome. Any population metric intended for cross-clock comparison therefore requires explicit orientation, clock-specific standardization, and reporting of the reference population.

A substantial literature links socioeconomic adversity to faster epigenetic ageing. Multicohort analyses have reported greater age acceleration in socially disadvantaged groups [9], and comparative work in the Multi-Ethnic Study of Atherosclerosis and Health and Retirement Study found robust associations for several adult socioeconomic domains, particularly with GrimAge, a DNA methylation estimator trained on mortality-related biomarkers, and DunedinPoAm, a DNA methylation measure of the pace of ageing [10]. A recent meta-analysis of 140 studies reported stronger social patterning for second- and third-generation clocks than for first-generation clocks [11]. Other studies have examined racialized, economic, and environmental conditions, socioeconomic circumstances across the life course, and mediation of disparities by education or neighborhood deprivation [12,13,14,15].

Building upon our previous systematic review and meta-analysis that established epigenetic age acceleration as a potentially modifiable public-health target and introduced the Modifiable Epigenetic Aging Burden Index (MEAB-Index) [16] and our ecological analysis of regional heterogeneity in healthy ageing and extreme longevity in Italy [17], the present study represents a natural methodological evolution of this line of research. Whereas the MEAB-Index quantifies the cumulative, potentially preventable biological-ageing burden attributable to modifiable risk factors, the current work shifts focus to how this epigenetic burden is distributed across an ordered socioeconomic hierarchy. Moving from modifiable burden to distributional inequality provides the conceptual foundation for a dedicated, population-level biological equity framework.

Despite these advances, existing studies and analytical approaches still leave an important measurement gap. Established socioeconomic-inequality measures, such as the Dissimilarity Index, the Concentration Index, and the Relative and Slope Indices of Inequality (RII and SII, respectively), summarize inequality in a single coefficient but primarily target means, rates, or categorized outcomes [18,19,20]. A regression slope can miss U-shaped or otherwise nonlinear patterns; a contrast between the most and least advantaged groups discards information from intermediate socioeconomic strata, and a measure based only on means can miss differences in dispersion, tails, or distributional shape. Conversely, an unsigned distributional distance can quantify separation without identifying whether the greater burden is concentrated among disadvantaged or advantaged groups. A single signed summary may also conceal heterogeneous or opposing patterns across the socioeconomic hierarchy and may therefore suggest little or no inequality when substantial distributional differences are present.

To address this gap, we developed the Epigenetic Coefficient (EGC) as a distribution-sensitive population-level profile rather than a single unqualified score. The EGC is designed to address four analytically distinct questions: How different are the EAA distributions across socioeconomic strata? In which direction does the inequality operate? How much of the observed pattern is captured by a linear socioeconomic gradient? And what is the overall population burden? The primary component, EGC-M, quantifies distributional separation across all ordered socioeconomic strata using weighted Wasserstein-1 distances, while EGC-O, EGC-S, and L provide complementary information on orientation, linear structure, and population level.

In plain terms, imagine measuring, for every person in a population, how much faster or slower their biological age runs relative to their chronological age, and arranging people from the most disadvantaged to the most advantaged socioeconomic position. A conventional analysis asks whether disadvantaged groups age faster on average. The EGC also compares the full spread of biological ageing within each group, because groups can have the same average yet differ markedly, for example when some disadvantaged individuals age much faster than others. It then reports separately how different the groups are, whether the extra burden falls mainly on the disadvantaged, how much of the pattern follows a simple gradient, and how high the overall burden is. Section 2.7 illustrates these ideas with two small numerical examples that can be checked by hand.

The objectives of this methodological study were therefore to: (i) define an estimand that uses all ordered socioeconomic strata and remains valid for negative, zero, and positive EAA values; (ii) distinguish distributional magnitude, direction, linear gradient, and population level; (iii) verify the point estimators independently in R (version 4.6.1; R Core Team, R Foundation for Statistical Computing, Vienna, Austria) and Python (version 3.14.5; Python Software Foundation, Wilmington, DE, USA); (iv) evaluate controlled null, directional, nonlinear, and distribution-only cases; and (v) establish an explicit pathway toward subsequent empirical and policy validation. The EGC is proposed as a candidate biological equity framework, not as an official United Nations indicator or an established measure of health inequity. Conceptually, it complements the MEAB-Index by shifting the focus from the modifiable burden of epigenetic ageing to its distribution across socioeconomic strata.

## 2. Materials and Methods

### 2.1. Study Design and Reporting Scope

This methodological development study comprised two linked workstreams. Workstream 1 specified the EGC estimand, verified its point estimators independently in R (version 4.6.1) and Python (version 3.14.5), and examined controlled structural cases and a small exploratory simulation. Workstream 2 conducted a structured evidence map to identify potentially equivalent or near-direct methods. No individual-level human cohort data were analyzed. The study therefore does not estimate a real-world EGC value, test biological transportability, or support causal or policy claims. Section 2.2, Section 2.3, Section 2.4, Section 2.5, Section 2.6, Section 2.7, Section 2.8 and Section 2.9 describe Workstream 1, and Section 2.10 describes Workstream 2.

The evidence was interpreted at four distinct levels. Algebraic results establish properties that follow from the definitions. Cross-language and fixed-dataset checks verify implementation consistency. The exploratory simulations illustrate behavior under selected data-generating conditions. Inferential calibration, empirical and biological validity, transportability, comparative utility, and policy performance remain unestablished. This hierarchy was fixed before empirical application so that observed cohort results could not influence the version 1.0 estimand.

The simulation component followed the ADEMP logic for aims, data-generating mechanisms, estimand, methods, and performance measures [21]. The present article reports the evidence sufficient for a methodological proof of concept. It does not constitute a formal statistical validation study. Large-scale operating-characteristic screening, real-cohort validation, normative threshold development, and policy-utility evaluation are prespecified subsequent stages of the EGC development programme rather than objectives of the present study.

### 2.2. Target Construct and Analysis Population

The target construct is the extent to which an oriented epigenetic ageing burden differs across prespecified, ordered socioeconomic strata within a defined population, period, tissue, assay, preprocessing pipeline, and clock. The corresponding estimand is a population-level functional of the stratum-specific individual-level distributions of epigenetic ageing burden. The primary unit is the individual. Aggregated stratum data can support descriptive extensions but do not preserve within-stratum distributions and are not automatically equivalent.

The estimand is defined at the individual level because the EGC is intended to compare empirical distributions of epigenetic ageing burden across socioeconomic strata. Individual-level observations preserve within-stratum heterogeneity, including differences in dispersion, tails, and distributional shape, that cannot be recovered from stratum-level means or other aggregate summaries.

For individual i and clock k, B_ik denotes an epigenetic ageing burden oriented so that larger values consistently indicate less favorable ageing. For comparisons across clocks, cohorts, or periods, the burden is standardized to a clock-specific external reference:

(1) $$Z_{ik}=\frac{B_{ik}-\mu_{ref,k}}{\sigma_{ref,k}}$$

The reference mean *μ*_ref,k and standard deviation *σ*_ref,k must be fixed and reported. If no defensible external reference is available, the EGC can be estimated in the native scale, but values from different clocks or cohorts must not be compared as if they shared a common metric [22]. Thus, standardization is a reporting and comparability requirement rather than a mathematical requirement for computing the EGC itself.

### 2.3. Socioeconomic Ordering and Weights

Socioeconomic position is represented by G ordered strata and ranks *R_g* in [0, 1], with 0 denoting maximum disadvantage and 1 maximum advantage. For ordinal categories, the preferred score is the weighted midpoint ridit: the cumulative population share below a category plus one-half of that category’s share. Ridits preserve order and encode relative population position without assuming equal distances between labels such as low, middle, and high education. They are nevertheless an estimand choice, not a neutral coding detail. If stratum shares change over time, contemporaneous ridits also change. Repeated comparisons must therefore prespecify either contemporaneous ridits, which target the current relative-rank distribution, or ridits fixed to a reference population, which preserve a common rank scale. The coding direction, reference, category order, and choice between contemporaneous and fixed ridits must be reported. Let *w_i* denote individual analysis weights and *p_g* the normalized weighted population share of stratum g; *w_i* = 1 when no analysis weights are required. Section 2.7 shows how this choice changes the gradient-based components in a simple example.

The socioeconomic ordering is treated as an analytic ordering variable rather than as a normative ranking of individuals. The choice of socioeconomic indicator, category boundaries, ordering convention, and weighting scheme must therefore be prespecified and reported in empirical applications.

### 2.4. EGC Architecture

The frozen scientific definition version 1.0 is the joint profile:

(2) $$EGC=({EGC}_{M},{EGC}_{O},{EGC}_{S};L)$$

EGC-MR is prespecified as a sensitivity analysis rather than a fifth component of the EGC profile.

The components are not interchangeable. EGC-M answers how different the distributions are; EGC-O asks whether a prevailing disadvantage-oriented direction is identifiable; EGC-S summarizes the linear mean gradient; and L reports the overall burden level needed to distinguish equitable improvement from levelling down. EGC-MR provides a rank-calibrated sensitivity analysis of the primary distributional magnitude and is not used to redefine or replace EGC-M. The components are intentionally complementary rather than alternative versions of the same statistic.

**Figure 1:**
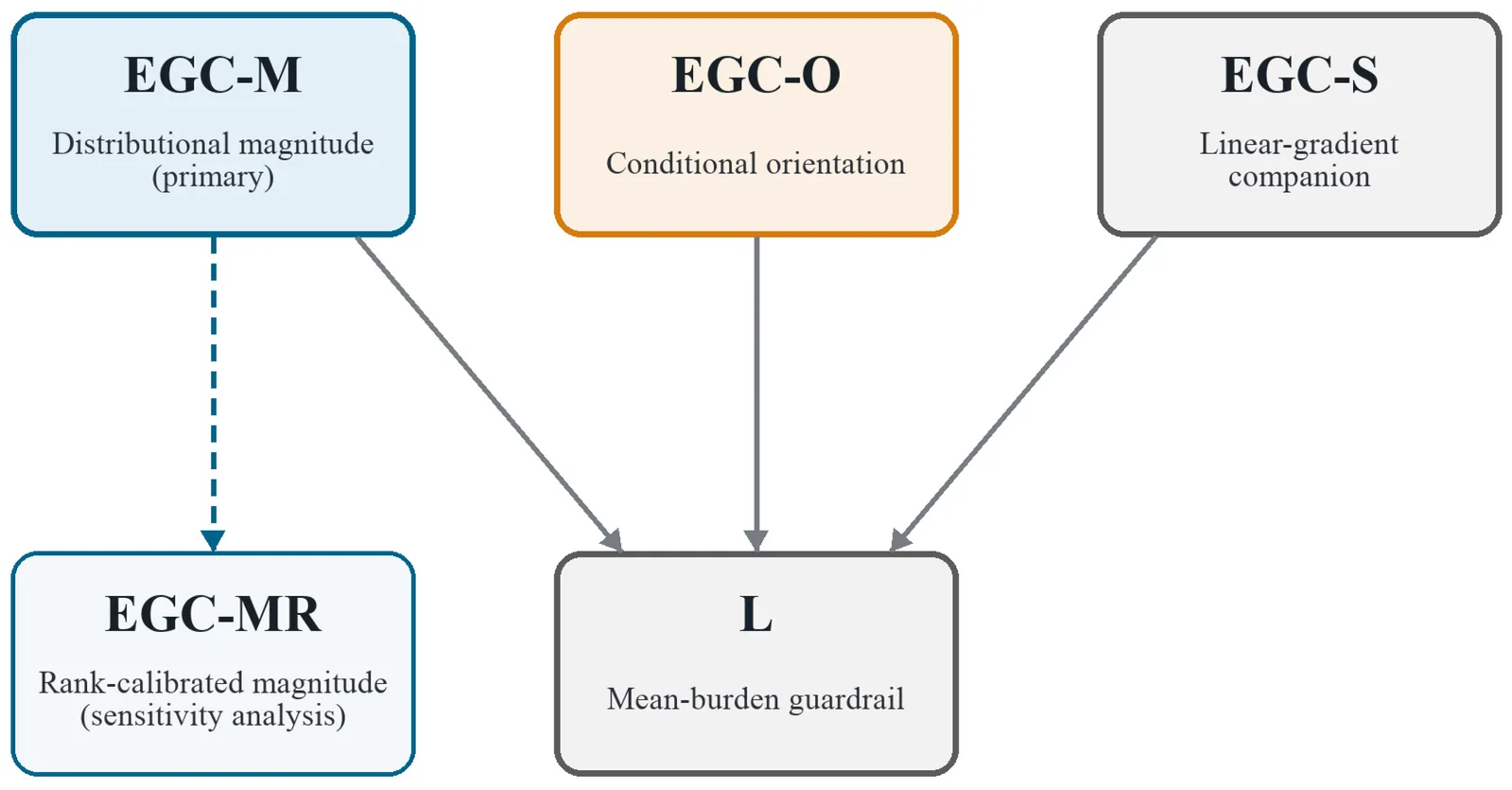
Architecture of the Epigenetic Coefficient. The primary EGC profile comprises EGC-M, EGC-O, EGC-S, and L. EGC-MR is a prespecified rank-calibrated sensitivity analysis of EGC-M and is not an additional component of the primary profile. EGC-O is interpreted only when its directional gate is satisfied. The dashed arrow indicates that EGC-MR is derived from EGC-M as a sensitivity analysis. Solid arrows indicate that EGC-M, EGC-O, and EGC-S are interpreted jointly with L, the population-level burden guardrail; they denote interpretation, not computation, as L does not enter the calculation of the other components.

Accordingly, the EGC is a structured profile rather than a scalar score. Each component has a prespecified estimand, interpretation, and limitation (Figure 1; Table 1).

#### 2.4.1. Primary Distributional Magnitude: EGC-M

Let $F_{g}$ denote the weighted empirical distribution of Z in stratum g. For each pair g < h, $W_{1}\left(F_{g},F_{h}\right)$ is the one-dimensional Wasserstein distance of order 1, equivalently the integral of the absolute difference between the weighted quantile functions [23,24]. The primary magnitude is

(3) $${EGC}_{M}=2\sum_{1\leq g<h\leq G}p_{g}p_{h}W_{1}(F_{g},F_{h})$$

Conceptually, EGC-M represents the population-weighted distributional separation generated by the socioeconomic hierarchy. Each pair of strata contributes according to the Wasserstein distance between their empirical EAA distributions, while the weighting scheme preserves the population structure of the target population. Thus, EGC-M incorporates information from all socioeconomic strata rather than relying only on the two extremes or on differences in stratum means.

EGC-M is non-negative and equals zero if and only if all positive-weight stratum distributions coincide. It is defined when EAA values are negative, zero, or positive; it does not divide by the sample mean; it is invariant to a common translation; and it scales proportionally under positive rescaling. The primary sample estimator is the raw empirical plug-in estimator. No finite-sample bias correction is incorporated into EGC-M version 1.0. Inference on distributional equality is instead performed through the prespecified permutation procedure.

#### 2.4.2. Rank-Calibrated Sensitivity: EGC-MR


(4) $${EGC}_{MR}=\frac{\sum_{1\leq g<h\leq G}p_{g}p_{h}d_{gh}W_{1}(F_{g},F_{h})}{\sum_{1\leq g<h\leq G}p_{g}p_{h}d_{gh}^{2}},d_{gh}=|R_{h}-R_{g}|$$

EGC-MR is a prespecified sensitivity measure that calibrates pairwise distributional separation to socioeconomic-rank distance. It does not replace EGC-M and is not part of the primary EGC profile.

Equation (4) is the weighted least-squares slope through the origin obtained by regressing the pairwise distance W_1_(*F_g,F_h*) on the rank distance *d_gh*, with pair weights *p_g p_h*. Under the linear location-shift model W_1_(*F_g,F_h*) = κd_gh, substitution into the numerator gives κ times the denominator, and EGC-MR = κ.

EGC-MR summarizes rank-distance calibration in a single scalar but does not by itself establish that pairwise separation increases monotonically with socioeconomic distance. Monotonicity must be assessed from the prespecified table or plot of pairwise W_1_ values against *d_gh*, together with the ordered stratum distributions. EGC-MR is therefore reported as a sensitivity parameter and cannot be used to redefine EGC-M after inspecting results.

#### 2.4.3. Conditional Orientation: EGC-O

For 1 ≤ g < h ≤ G, where stratum g is more disadvantaged, define the weighted probability-of-superiority contrast as follows. Ties contribute zero to the signed contrast; equivalently, each tie receives one-half weight in the probability-of-superiority representation:

(5) $$\delta_{gh}=P(Z_{g}>Z_{h})-P(Z_{g}<Z_{h})$$

(6) $$a_{gh}=p_{g}p_{h}(R_{h}-R_{g}),1\leq g<h\leq G$$

(7) $$A=\sum_{1\leq g<h\leq G}a_{gh}\delta_{gh},D=\sum_{1\leq g<h\leq G}a_{gh}|\delta_{gh}|,{EGC}_{O}=\frac{A}{D}\;\mathrm{for}\;D>0$$

Positive values indicate a prevailing greater burden in disadvantaged strata and negative values the reverse. A value near zero can reflect conflicting pairwise directions and is not evidence of equality. If D = 0, orientation is not identified and remains missing. EGC-O is conditional on distributional separation and is not an independent measure of inequality magnitude. For version 1.0 reporting, orientation is described as directionally supported only when the global EGC-M test rejects equality, the uncertainty interval for A excludes zero, shape diagnostics support a directional summary, and the sign is stable across prespecified sensitivities. This gate is a proposed prespecified reporting rule, not an empirically calibrated classifier. Its false-positive rate, sensitivity, and stability under sparse, unbalanced, clustered, or complex crossing distributions remain to be quantified.

Accordingly, “orientation not identified” and “no socioeconomic inequality” are distinct outcomes and must never be coded interchangeably.

#### 2.4.4. Linear Companion and Level Guardrail


(8) $$E(Z_{i}|R_{i},X_{i})=\alpha +\beta R_{i}+\theta^{T}X_{i},{EGC}_{S}=-\beta$$

EGC-S summarizes the linear component of mean burden along the prespecified SES rank and is secondary to the distributional estimand. With ordinal SES, linearity refers to the chosen rank score, usually midpoint ridits, rather than to equal spacing between category labels. Ordered stratum summaries and monotonicity diagnostics must accompany EGC-S so that a U-shaped or otherwise non-monotone pattern is not reduced to a misleading slope.

With R spanning 0 to 1, EGC-S is the predicted burden difference between the theoretical extremes of disadvantage and advantage. It is positive when disadvantage is associated with greater burden. The weighted population mean, reported as the level guardrail, is

(9) $$L=\sum_{g=1}^{G}p_{g}\mu_{g}$$

where μ_g denotes the weighted mean for stratum g. L is not an inequality measure and is not incorporated into EGC-M, EGC-O, or EGC-S. It is reported as a contextual level parameter.

A decline in EGC-M is favorable only when interpreted jointly with L and the full stratum profile. A reduction in distributional inequality may occur through improvement in disadvantaged strata, deterioration in advantaged strata, or both; EGC-M alone cannot distinguish these mechanisms. L is therefore a guardrail against interpreting “less inequality” as synonymous with “better population health”.

### 2.5. Inference and Diagnostics

The global null hypothesis is the equality of all positive-weight stratum distributions. The version 1.0 permutation procedure is intended for independent observations whose socioeconomic labels are exchangeable under the null, either unconditionally or within prespecified exchangeability blocks. Under those conditions, labels are permuted and EGC-M is recalculated. Monte Carlo *p*-values use the plus-one correction [25]:

(10) $$\hat{p}=\frac{1+\sum_{b=1}^{B}I[T_{b}^{perm}\geq T_{obs}]}{B+1}$$

The observed EGC-M is the distributional effect-size component, whereas the permutation distribution assesses compatibility with the global equality null. Computing a weighted EGC-M does not make unrestricted label permutation design-consistent. If weights are informative, observations are clustered, or data arise from stratified or multistage sampling, valid inference requires a design-specific randomization, restricted permutation, cluster resampling, or replicate-weight procedure. These extensions have not been statistically calibrated within EGC v1.0. In such settings, EGC-M may be reported descriptively, but the unrestricted permutation *p*-value must not be presented as confirmatory.

The empirical distance remains positive under a population null; permutation inference addresses this sampling noise without assuming that it can be subtracted additively from the observed distance.

For independent observations, the current recommended uncertainty procedure is a nonparametric bootstrap stratified by socioeconomic stratum, with the analysis weights carried with sampled observations. Clustered samples require cluster-level resampling, and complex surveys require design-consistent replicate weights or another survey-specific procedure; these alternatives remain outside the validated scope of version 1.0. Because Wasserstein functionals and equality-boundary problems can be non-regular at or near exact equality, a numerical bootstrap interval near zero is not assumed to have nominal coverage automatically [24,26]. When regularity or design assumptions are doubtful, authors should report the point estimate, permutation result only if exchangeability is defensible, pairwise distances, effective stratum sizes, weighted quantiles, and shape diagnostics, and should label interval-based orientation as exploratory or inconclusive.

These diagnostics identify situations in which a scalar directional summary may be incomplete or misleading and are part of the interpretation of an empirical EGC rather than optional post hoc checks.

EGC-M and EGC-O are marginal distributional summaries for the defined target population in version 1.0. EGC-S can include prespecified covariates through its regression formulation. An adjusted distributional analysis would require standardized or model-based counterfactual stratum distributions, a clearly defined target population, and resampling that accounts for estimation of the adjustment model. Such an analysis changes the estimand and its exchangeability conditions and is treated as a future extension rather than an interchangeable implementation of the frozen marginal EGC-M and EGC-O.

### 2.6. Computational Verification

Point estimators were implemented independently in R (version 4.6.1) and Python (version 3.14.5). Twenty fixed datasets containing 5006 observations covered exact nulls, positive and inverse gradients, U-shaped and inverse-U patterns, scale and tail differences with equal means, crossing distributions, unequal weights, unbalanced and sparse strata, ties, heavy tails, contamination, and increased category granularity. Agreement was assessed for seven prespecified profile-level quantities across the 20 datasets, yielding 140 component-level comparisons, together with 1095 pairwise intermediate quantities under the prespecified absolute-plus-relative tolerance:

(11) $$\left|x_{R}-x_{Python}|\leq 10^{-10}+10^{-10}|x_{Python}\right|$$

The cross-language exercise was designed to verify implementation consistency, not to establish statistical validity or operating characteristics.

Two six-observation worked examples supplied exact expected values and are presented below to make the component interpretations transparent. A separate exploratory pilot used three controlled conditions—an exact null, a positive gradient, and a negative gradient—with *N* = 2500 and five replications per condition. Each replication used 499 permutations and 499 bootstrap resamples. The pilot was a computational sanity check, not an operating-characteristic study.

### 2.7. Worked Example

Consider three equally sized SES strata, each containing two standardized burden values. Higher values indicate less favorable ageing. In Example A, the values form an exact location-shift gradient; in Example B, all stratum means are equal but dispersion decreases from the disadvantaged to the advantaged stratum (Table 2).

**Table 2:** Six-observation worked examples.

SES Stratum	Equally Spaced Score	Midpoint Ridit	Example A: Burden Values	Example B: Burden Values	Reading (Example A)
Disadvantaged	0.0	0.167	1, 2	−2, 2	Highest burden
Middle	0.5	0.500	0, 1	−1, 1	Intermediate burden
Advantaged	1.0	0.833	−1, 0	0, 0	Lowest burden

Example A yields EGC-M = 0.889, meaning that the population-weighted average pairwise separation of the three complete distributions is 0.889 standard-deviation units. EGC-MR = 2 because each unit of SES-rank distance corresponds to a two-unit location shift. EGC-O = 1 because every pairwise comparison points toward greater burden in the disadvantaged group. EGC-S = 2 gives the same extreme-to-extreme mean gradient in this exactly linear case, and L = 0.5 reports the overall mean burden. Agreement of EGC-MR and EGC-S is a property of this constructed location-shift example, not a general identity.

Example B shows what a mean-based summary cannot detect. EGC-M is again 0.889, so the overall distributional separation equals that of Example A. However, EGC-S = 0 and L = 0, because all stratum means are zero, and EGC-O is not identified (D = 0), because within each pair of strata the favorable and unfavorable comparisons cancel out. EGC-MR remains 2, confirming that it calibrates magnitude to rank distance and carries no directional information. The two examples therefore share the same distributional magnitude but differ in structure: a directional gradient in Example A and a dispersion difference without a prevailing direction in Example B. Only the full profile distinguishes them. In Example B, the appropriate report is a distributional separation with orientation not identified, not an absence of inequality.

Equally spaced scores are used above for arithmetic transparency. Replacing them with the recommended midpoint ridits (1/6, 1/2, and 5/6) in Example A leaves EGC-M (0.889) and L (0.5) unchanged, because these components do not use ranks, and EGC-O remains 1 because all pairwise contrasts still point in the same direction. EGC-MR and EGC-S, however, both become 3: the observed strata now span two-thirds of the rank scale, so the same mean differences are expressed per unit ridit. The choice of rank score is therefore part of the estimand for the gradient-based components and must be prespecified (Section 2.3).

### 2.8. Reproducibility and Version Control

The construct, direction convention, formulas, reporting rules, and point estimators are frozen as EGC scientific definition v1.0. Reference implementations, expected outputs, random seeds, and a SHA-256 manifest are available in the public repository (see Data Availability Statement).

The frozen specification is intended to make the estimand auditable and prevent outcome-dependent modification during subsequent empirical testing.

Undefined orientation is never converted to zero. Any future change to the frozen estimand requires a dated, versioned amendment separated from affected outcomes.

### 2.9. Minimum Reporting Standard

The minimum reporting requirements for an empirical EGC result are summarized below (Table 3).

### 2.10. Structured Precedent Mapping and Novelty Audit

Workstream 2 was designed to determine whether an equivalent coefficient already existed and was conducted independently of the EGC specification and verification described above. A retrospective corpus of 307 PDF records, previously assembled from four conceptual search streams, was normalized and deduplicated by title, DOI, publication identity, and version relationships. This process produced 238 canonical publications. Each publication was classified for human biological ageing, DNAm-based EAA, ordered socioeconomic strata, distributional inequality estimand, and presence of a named synthetic coefficient designed for the socioeconomic distribution of EAA.

An operational P0 equivalent required all of the following: human DNAm-EAA; ordered socioeconomic strata; an explicit distributional inequality estimand; and a named synthetic coefficient or index developed for that purpose. P1 denoted a near-direct methodological precedent that addressed several, but not all, P0 criteria. P2 denoted an empirical association or socioeconomic-gradient study; P3 denoted methodological or measurement context; and P4 denoted broader contextual evidence. Records outside the conceptual or methodological scope were classified as not pertinent. All eight P1 publications underwent full-text claim-level audit. Screening and P0–P4 classification were performed by P.M. and verified by S.M.A.; disagreements were resolved by discussion. The source corpus was assembled between 8 and 16 July 2026.

The retrospective corpus did not retain record-level database, query, or original search-date provenance. It is therefore reported as a structured developmental evidence map rather than a PRISMA systematic review. The reconstructed search assets comprise five operational query families formatted for PubMed, Scopus, Web of Science, and Europe PMC (20 platform-query combinations). The four conceptual streams corresponded to four questions (Q1–Q4: socioeconomic associations with EAA; formal inequality measures; reliability and comparability of clocks; ageing biomarkers as equity or SDG indicators). Q2 was operationalized as two query families, Q2A for DNAm-based EAA and Q2B for non-epigenetic biological age, so that a non-epigenetic result could not be mistaken for a direct precedent (Document S1, Section S1.2). They were reconstructed after the corpus had been assembled to document the conceptual search and were not used to generate the corpus. The strings were constructed on 30 July 2026: the PubMed and Europe PMC versions were executed to confirm syntactic executability, and the Scopus and Web of Science versions were archived in fully expanded form. The integrity of all 20 archived strings was verified against a SHA-256 manifest on 10 August 2026; the searches were not re-run to update the corpus. The complete strings, eligibility and deduplication rules, classification definitions, canonical-publication registry, and P1 audit are supplied in the Supplementary Materials (Document S1 and Table S1). Because the reconstructed strings cannot be linked retrospectively to each of the 307 physical records, database-specific yields, recall, and exhaustive literature-wide priority are not claimed.

## 3. Results

Results are reported in the order of the two workstreams: Section 3.1, Section 3.2 and Section 3.3 describe computational verification and structural behavior of the EGC, and Section 3.4 reports the evidence-map findings.

### 3.1. EGC Computational Verification

All prespecified cross-language comparisons passed. R and Python agreed on 140 component-level quantities and 1095 pairwise quantities, for 1235/1235 successful comparisons. No defined/undefined status diverged. The maximum absolute difference was 3.29 × 10^−14^, observed for a probability-of-superiority contrast in a dataset containing many ties. The verification also confirmed the expected structural properties of the frozen estimators, including cross-language agreement within the prespecified numerical tolerance, translation invariance, positive-scale equivariance, detection of distributional differences despite equal means, and preservation of undefined orientation when directional information was not identifiable (Table 4).

### 3.2. Structural Cases and Worked Example

The fixed-dataset suite demonstrated the distinct information captured by the EGC components. In a deterministic positive gradient, EGC-S = 0.40, EGC-M = 0.128, EGC-MR = 0.40, and EGC-O = 1. In an equal-mean scale-gradient dataset, EGC-S and the difference in stratum means were zero, whereas EGC-M remained positive, demonstrating sensitivity to distributional differences beyond location. In a crossing-distribution dataset, stratum means were zero and EGC-M = 0.576, whereas orientation was not identifiable. These cases illustrate the complementary information provided by the EGC profile and were designed as mathematical stress tests rather than representations of biological data.

The two six-observation examples in Table 2 provide an intuitive numerical reading of the same architecture. The exact location-shift structure of Example A produces concordant EGC-MR and EGC-S values, whereas Example B, together with the equal-mean and crossing cases above, shows why the distributional magnitude and orientation cannot be replaced by the linear companion.

### 3.3. Exploratory Simulation Proof of Concept

The N = 2500 pilot completed 15 prespecified replications without computational or bootstrap failures. In the five exact-null samples, the global EGC-M test was non-significant, and the proposed orientation gate remained closed. In the ten gradient samples, the global test was significant, and EGC-S and the orientation contrast had the expected sign. These counts describe the selected runs only; they do not estimate error rates, power, sensitivity, specificity, or coverage.

**Figure 2:**
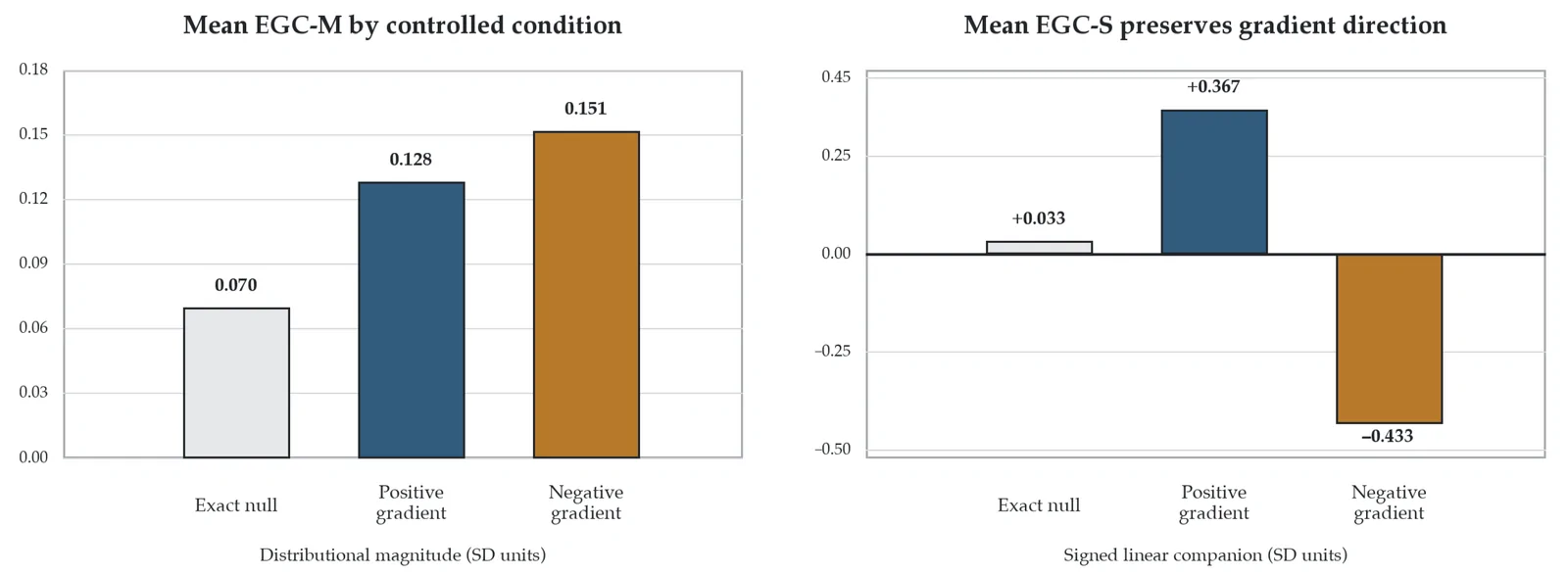
Exploratory simulation proof of concept. Bars show mean point estimates across five replications per controlled condition at *N* = 2500. The exact-null EGC-M sample estimate remains positive because it is an empirical distance; the permutation test, not subtraction, determines whether the observed separation exceeds null noise.

Mean EGC-M was 0.0697 under the exact-null sample, 0.1279 under the positive gradient, and 0.1515 under the negative gradient. Mean EGC-S values were +0.0326, +0.3668, and −0.4335, respectively (Figure 2; Table 5).

**Table 5:** Exploratory *N* = 2500 simulation results (five replications per condition).

Condition	Mean EGC-M	Mean EGC-S	Global *p* < 0.05	Orientation Gate	Correct Sign
Exact null	0.0697	+0.0326	0/5	0/5	Not applicable
Positive gradient	0.1279	+0.3668	5/5	5/5	5/5
Negative gradient	0.1515	−0.4335	5/5	5/5	5/5

Note: Correct-sign proportions are descriptive only and are not estimates of classification accuracy, sensitivity, or power.

### 3.4. Evidence-Mapping Results

Having described the computational behavior of the EGC, we now turn to the second workstream, which assessed whether equivalent or near-direct methods existed in the literature. The evidence-mapping workstream began with 307 physical PDF records and resolved them to 238 canonical publications. Screening retained 160 publications as relevant to the conceptual or methodological scope and excluded 78. Among the 160 included publications, 106 were empirical, 45 provided methodological or measurement context, and nine were mapping or review sources. The hierarchy comprised 117 P2, 23 P3, 11 P4, eight P1, no P0, and one included publication that could not be assigned to P0–P4; this record and the reason for non-assignment are identified in the canonical-publication registry (Table S1). The categories therefore account for all 160 included publications (Table 6).

The eight P1 publications were the closest precedents [14,15,27,28,29,30,31,32]. They used regression attenuation, mediation, Fairlie or Kitagawa–Blinder–Oaxaca decomposition, mixture modeling, or intersectional variance partitioning. None combined DNAm-EAA, ordered socioeconomic strata, a complete-distribution inequality estimand, and a named coefficient developed for that purpose. Table 7 lists each P1 precedent and the criterion preventing P0 classification; the claim-level audit is supplied in Table S1. This finding supports methodological distinctiveness within the examined corpus, not universal priority (Table 6 and Table 7).

**Table 6:** Structured evidence-map disposition and novelty result.

Item	Result	Interpretation
Physical PDF records	307	Retrospective source corpus
Canonical publications	238	Deduplicated publication unit
Included/excluded	160/78	Conceptual–methodological relevance
Near-direct P1 precedents	8	Full-text claim-level audit completed
Equivalent P0 coefficients	0	None within the frozen corpus
Query strings archived	20/20	Hash integrity checked; 10/20 executed for syntax (PubMed, Europe PMC); corpus not updated

**Table 7:** Near-direct P1 precedents and reason for non-equivalence.

Reference	Study	Closest Method	Why Not P0
[27]	Alonso-Perez et al., 2026	Clinical PhenoAge; intersectional variance partitioning	Not DNAm-EAA and not an ordered SES distribution coefficient
[28]	Knobel et al., 2025	Clinical PhenoAgeAccel; social-vulnerability mixture	Not DNAm-EAA and not a coefficient of EAA inequality
[29]	Yannatos et al., 2023	Regression and mediation of cognitive and functional disparities	Does not summarize EAA distributions across ordered SES strata
[30]	Boen et al., 2023	Kitagawa-Blinder-Oaxaca decomposition of clinical biological age	Not DNAm-EAA and not an ordered SES coefficient
[31]	Yannatos et al., 2023	Decomposition of racial differences in GrimAge and DunedinPoAm	Targets a racial gap, not the EAA distribution along ordered SES strata
[14]	Chen et al., 2024	Regression and mediation of EAA disparities	No distributional inequality coefficient across ordered SES strata
[15]	Fiorito et al., 2022	Epigenetic clocks as mediators of educational mortality inequalities	Mortality inequality is the target; EAA is a mediator
[32]	Abugroun et al., 2026	Staged decomposition of binary accelerated GrimAge	Uses a binary outcome and racial gap rather than continuous EAA distributions across ordered SES

## 4. Discussion

### 4.1. Interpretation of Findings

The principal contribution of this study is not simply the introduction of another summary measure of socioeconomic gradient, but the specification of a distribution-sensitive framework for measuring inequality in epigenetic ageing. Existing approaches can quantify associations, contrasts, mediation, decomposition, or linear socioeconomic gradients, but they do not simultaneously preserve distributional information on EAA across an ordered socioeconomic hierarchy while separating magnitude, direction, linearity, and population-level burden. The EGC was designed to address this specific measurement gap.

The frozen EGC v1.0 therefore provides a joint profile comprising four analytically distinct elements: EGC-M quantifies distributional separation across socioeconomic strata; EGC-O evaluates whether a prevailing disadvantage-oriented direction can be identified; EGC-S captures the linear component of the socioeconomic pattern; and L provides the population-level burden required to distinguish genuine improvement from levelling down. These components are intentionally non-interchangeable and should be interpreted jointly rather than collapsed into a single scalar summary.

The current evidence supports a limited set of claims. The definitions establish algebraic properties such as non-negativity of EGC-M and equality EGC-M = 0 when all positive-weight stratum distributions coincide. Independent implementations establish numerical reproducibility of the point estimators. Controlled cases and the small pilot illustrate structural behavior under selected scenarios. They do not establish inferential calibration, empirical sensitivity or specificity, biological validity, transportability, longitudinal responsiveness, or policy utility.

### 4.2. Novelty and Contribution to the Literature

Prior studies have established social gradients in epigenetic ageing and have used regression, group contrasts, mediation, decomposition, and variance partitioning [9,10,11,12,13,14,15]. The EGC addresses a different estimand: the weighted distributional separation of EAA across all ordered socioeconomic strata, coupled with a conditional direction and a population-level guardrail. The distinction is therefore not primarily one of statistical complexity, but of the target quantity being measured: the EGC is designed to retain distributional information that conventional mean- or slope-based summaries may discard.

This positioning also extends a broader line of population-health research in which heterogeneity in ageing and longevity is treated as a multidimensional population phenomenon rather than as a single outcome. Our recent ecological analysis of healthy ageing and extreme longevity across Italy demonstrated substantial regional heterogeneity in demographic ageing and longevity profiles and highlighted the importance of contextual differences in shaping population-level ageing patterns [17]. The EGC builds on this population-level perspective by moving from the description of between-population heterogeneity to a formal distribution-sensitive framework for quantifying socioeconomic inequality in an underlying biological-ageing phenotype.

Within the examined 238-publication corpus, no equivalent P0 coefficient was identified. This finding supports a claim of methodological distinctiveness within the searched corpus, rather than a universal claim of priority. The defensible wording is therefore “to our knowledge” and “within the searched corpus,” not an unconditional claim that no related method exists anywhere in the literature.

The EGC is not a renamed Gini coefficient, Dissimilarity Index, Concentration Index, or Relative or Slope Index of Inequality. These measures remain important comparators [18,19,20]. The Dissimilarity Index summarizes redistribution between groups but does not inherently use SES ordering and generally requires categorization of the outcome. Concentration-based measures provide a signed single-number summary of rank-related inequality, but their interpretation and normalization depend on the outcome scale and mean; standard formulations are problematic for signed EAA values with no stable ratio-scale zero. Relative and Slope Indices of Inequality summarize a modeled gradient across ridit rank and provide direction, but primarily target a mean, rate, or risk contrast. EGC-M instead targets complete-distribution separation without division by the mean, while EGC-O and EGC-S report orientation and the rank gradient separately. This separation sacrifices the convenience of one coefficient but prevents opposing directions, nonlinearity, or shape differences from being hidden in a single signed value (Table 8).

Unsigned distributional separation is not interpreted as inequity by itself. It signals that the stratum distributions differ, including in dispersion or tails, and prompts inspection of EGC-O, ordered stratum summaries, and substantive context. If no stable direction is identifiable, the appropriate result is distributional separation with inconclusive orientation, not a claim of disadvantage-oriented inequity. Whether this information improves decisions beyond established signed measures is an empirical question reserved for head-to-head validation.

### 4.3. Potential Relevance to SDG 3 and SDG 10

SDG 3 concerns health and well-being, including prevention of premature non-communicable disease mortality, while SDG 10 concerns the reduction in inequalities [1,2,3]. The EGC is conceptually positioned at their intersection: it asks whether a molecular marker of ageing is distributed unequally along a socioeconomic hierarchy. This makes the framework potentially useful as a candidate biological equity framework within research or surveillance systems that already include conventional health and social indicators. This perspective is consistent with a broader prevention-oriented approach to biological ageing developed in our previous work. In a recent systematic review and meta-analysis, we synthesized evidence linking epigenetic age acceleration to modifiable environmental, behavioral, metabolic, and social determinants and developed the Modifiable Epigenetic Aging Burden Index (MEAB-Index) as a framework for quantifying potentially preventable biological-ageing burden [16]. Whereas the MEAB-Index addresses the potential modifiability and cumulative burden of determinants associated with EAA, the EGC addresses a different question: how the resulting biological-ageing burden is distributed across socioeconomic strata. These frameworks are therefore complementary, linking the concepts of modifiable biological ageing, population burden, and distributional equity without implying that either metric is an established policy indicator.

However, biological measurements can amplify inequity if access, ancestry, tissue composition, assay platforms, or data governance are ignored. The EGC should therefore supplement, never replace, social and health indicators. Formal KPI adoption would require representative sampling, validated reference distributions, measurement invariance, stakeholder acceptability, cost assessment, interpretability thresholds, longitudinal responsiveness, and evidence that the EGC changes decisions beneficially and equitably. The EGC should therefore be understood at this stage as a research-level biological equity framework with potential future relevance to health-equity surveillance, rather than as a replacement for established socioeconomic or health inequality indicators. No United Nations endorsement is claimed.

### 4.4. Strengths and Limitations

Strengths include construct-first development; explicit separation of magnitude, direction, slope, and level; mathematical handling of negative and near-zero EAA; use of all socioeconomic strata and population weights; preservation of undefined orientation; independent R and Python implementations; exact and stress-case datasets; frozen formulas and manifests; and an explicit restriction of causal, empirical, normative, or policy claims to those supported by the current evidence.

The most important limitation is the absence of individual-level real-cohort data. The study establishes mathematical specification, computational reproducibility, and structural proof of concept only. The five-replication pilot cannot estimate type-I error, power, bias, interval coverage, classification accuracy, or robustness across clocks or complex designs. Likewise, the orientation gate is a proposed prespecified reporting rule; its false-positive rate, sensitivity, and stability have not been quantified. The unrestricted permutation and stratified bootstrap procedures also require assumptions that may not hold with informative weights, clustering, covariate-standardized distributions, or multistage surveys. The EGC is descriptive rather than normative: distributional inequality must not automatically be interpreted as inequity, injustice, or avoidable inequality. The socioeconomic hierarchy, burden orientation, reference population, and weighting scheme are analytic choices that must be prespecified and reported.

### 4.5. Future Validation Programme

The next empirical study should apply the frozen EGC v1.0 formula to a prespecified individual-level cohort containing EAA, an ordered socioeconomic measure, relevant sampling weights, and technical and demographic covariates. Validation should proceed sequentially rather than through immediate application to a single observational dataset. First, large-scale simulation studies should quantify type-I error, power, bias, interval coverage, sensitivity to sparse strata, and robustness to alternative distributional structures. Second, a development cohort should be used for empirical feasibility and construct validation. Third, an independent cohort should provide external validation, including clock-specific sensitivity analyses and assessment of transportability. Fourth, prespecified comparisons with established inequality measures should determine whether the EGC provides incremental distributional information. Finally, longitudinal and policy-oriented studies should assess responsiveness, interpretability, stakeholder acceptability, and potential utility for health-equity surveillance. Real-data application is deliberately separated from the current methodological paper so that outcomes cannot influence the formula.

### 4.6. Methodological Positioning of the EGC

The EGC should be viewed as a distributional complement to, rather than a replacement for, established measures of socioeconomic inequality. Together with complementary frameworks addressing the modifiable determinants and population burden of biological ageing, such as the MEAB-Index [16], the EGC may contribute to a broader prevention-oriented architecture in which biological ageing is considered both a modifiable health target and a dimension of population-health equity. Its distinctive target is the distribution of epigenetic ageing burden across an ordered socioeconomic hierarchy. Accordingly, the EGC is most informative when distributional heterogeneity is substantively relevant and when mean-based summaries may obscure important differences. In empirical applications, reporting EGC-M alongside EGC-S, EGC-O, L, and conventional inequality measures will allow investigators to determine whether the distribution-sensitive framework contributes information beyond established approaches.

## 5. Conclusions

The Epigenetic Coefficient (EGC) introduces a candidate distribution-sensitive framework for characterizing socioeconomic inequality in epigenetic ageing. Its central methodological contribution is to use distributional information from the full empirical distributions of epigenetic ageing burden while analytically separating magnitude, direction, linear gradient, and population-level burden.

The EGC v1.0 estimand is mathematically specified, version frozen, and independently reproduced in R and Python. Controlled scenarios demonstrate coherent behavior by construction and in selected stress cases. These results do not establish the statistical calibration of the proposed permutation, bootstrap, or directional procedures, nor empirical, biological, causal, normative, comparative, or policy validity.

The EGC is therefore best positioned at this stage as a candidate research framework for biological health equity. Its future value will depend on independent methodological verification, systematic comparison with established inequality measures, large-scale operating-characteristic studies, and application to independent individual-level cohorts. Only after these steps should its potential contribution to health-equity surveillance or SDG-related monitoring be evaluated.

## Figures and Tables

**Table 1:** Frozen components of the EGC version 1.0.

Component	Definition	Primary Interpretation	Mandatory Qualification
EGC-M	Weighted pairwise W_1_ distributional magnitude	Average separation among stratum distributions	Non-negative; no direction by itself
EGC-MR	Weighted least-squares calibration of pairwise W_1_ to SES-rank distance	Distributional separation per unit rank distance under the specified projection	Sensitivity; does not prove monotonicity or replace EGC-M
EGC-O	A/D probability-of-superiority orientation	Prevailing direction of burden	Conditional; undefined if D = 0
EGC-S	-β from burden on SES rank	Linear component along the prespecified SES rank	May miss non-monotone or distribution-only inequality
L	Weighted mean burden	Population-level burden guardrail	Required to avoid interpreting levelling down as improvement

**Table 3:** Minimum information required for an empirical EGC result.

Domain	Required Report
Population	Cohort, country, period/wave, eligibility, tissue and analytic N
Epigenetic burden	Clock/version, assay, preprocessing, EAA definition and orientation
Standardization	Reference population, *μ*_ref and *σ*_ref, or explicit native-scale limitation
Socioeconomic hierarchy	Domain, categories, order, ridits/ranks and stratum shares
Design	Weights, clustering, effective sample sizes, missing-data handling, and the inferential procedure justified for the design
EGC profile	EGC-M with point estimate and inference only when assumptions are met; EGC-MR; EGC-O or inconclusive; EGC-S; L
Diagnostics	Stratum distributions, means/quantiles, pairwise W_1_ and *δ*, W_1_-versus-rank-distance display, and shape and sparse-stratum checks
Reproducibility	Formula/software versions, resampling counts, seeds and analysis code

**Table 4:** Independent R–Python verification of EGC point estimators.

Verification Level	Comparisons	Passed	Maximum Absolute Difference
EGC profile components	140	140	1.02 × 10^−14^ for EGC-S
Pairwise quantities	1095	1095	3.29 × 10^−14^ for δ_gh
Total	1235	1235	3.29 × 10^−14^

**Table 8:** Conceptual comparison with established socioeconomic-inequality measures.

Measure	Primary Target	Use of SES Ordering	Direction	Key Qualification
Dissimilarity Index	Redistribution between groups	No inherent SES rank	Usually unsigned	Requires outcome categorization; does not compare full continuous distributions
Concentration Index	Rank-related inequality in an outcome	Uses relative SES rank	Signed single coefficient	Normalization and interpretation depend on outcome scale and mean
RII/SII	Modeled extreme-to-extreme relative or absolute gradient	Uses ordered rank/ridit	Signed single coefficient	Model-based mean/rate/risk contrast; sensitive to functional form
EGC profile	Complete-distribution separation plus separate orientation, rank gradient, and level	Uses ordered strata and prespecified ranks	Magnitude and direction reported separately	No single score; inferential and incremental validity remain to be established

## Data Availability

No individual-level human data were generated or analyzed. All datasets used for mathematical verification and simulation were controlled or simulated specifically for this methodological study. The R and Python code, controlled datasets, expected outputs, and pilot scripts with seeds are openly available at https://github.com/EGC-epigenetic-coefficient/epigenetic-coefficient (release EGC v1.0.0), accessed on 14 September 2026. The search strategies and the publication registry are provided in the Supplementary Materials (Document S1 and Table S1). Copyrighted article PDFs are not redistributed.

## Supplementary Materials

The following supporting information can be downloaded at https://www.mdpi.com/article/10.3390/life16101720/s1. Document S1: Search strategies, deduplication and eligibility rules, and P0–P4 classification definitions for the structured precedent mapping (Workstream 2); Table S1: Canonical-publication registry of the structured precedent mapping (Workstream 2), including the claim-level audit of the eight P1 near-direct precedents. The R and Python implementations of EGC v1.0, the controlled datasets with expected outputs and the cross-language comparison script, the pilot script with seeds, and a SHA-256 manifest are openly available at https://github.com/EGC-epigenetic-coefficient/epigenetic-coefficient (release EGC v1.0.0), accessed on 14 September 2026. References [14,15,27,28,29,30,31,32] are cited in the Supplementary Materials.

## Abbreviations

The following abbreviations are used in this manuscript:

DNAm	DNA methylation
EAA	Epigenetic age acceleration
EGC	Epigenetic Coefficient
EGC-M	Primary distributional magnitude
EGC-MR	Rank-calibrated magnitude sensitivity
EGC-O	Conditional orientation
EGC-S	Linear-gradient companion
KPI	Key performance indicator
SES	Socioeconomic status or position
SD	Standard deviation
SDG	Sustainable Development Goal
W1	Wasserstein distance of order 1
