# Supplementary material for: Beyond the Socioeconomic Gradient: The Epigenetic Coefficient (EGC) as a Distribution-Sensitive Framework for Measuring Inequality in Epigenetic Ageing
Source: Life (Basel). 2026 Oct 15;16(10):1720. doi: 10.3390/life16101720 (PMC13706368; doi:10.3390/life16101720)
Supplement: Supplementary file 1 [file life-16-01720-s001.zip › life-4583845-supplementary.pdf]

Supplementary Materials

Document S1. Search strategies, deduplication and eligibility rules, and P0–P4 classification definitions for the structured precedent mapping (Workstream 2)

Epigenetic Coefficient (EGC). This document supports Section 2.10 of the main text. The canonical-publication registry, screening decisions, precedent classes and the P1 claim-level audit are provided separately in Table S1.

S1.1. Status and chronology of the search assets

The evidence map was built on a retrospective corpus of 307 PDF records. Publisher download stamps and file-creation dates show that the source PDFs were obtained between 8 and 16 July 2026. The corpus did not retain record-level provenance (database, query string, or original search date) for any of the 307 records; the evidence map is therefore reported as a structured developmental evidence map and not as a PRISMA systematic review.

The search assets reported below were reconstructed after the corpus had been assembled. They comprise four conceptual questions translated into five operational query families (Q1, Q2A, Q2B, Q3, Q4) for four platforms (PubMed, Scopus, Web of Science Core Collection, Europe PMC preprints), giving 20 platform–query combinations. Their status is summarized in Table S1.1.

Step	Date	Scope
Corpus assembly (PDF acquisition)	8–16 July 2026	307 PDF records
String construction and technical execution	30 July 2026	PubMed and Europe PMC strings executed to confirm syntactic executability; Scopus and Web of Science strings fully expanded and archived but not executed
Deduplication and screening	1–10 August 2026	307 records → 238 canonical publications; all 238 screened
Integrity check of the archived strings	10 August 2026	20/20 UTF-8 strings matched the SHA-256 manifest; 20/20 ASCII copies present

Table S1.1. Chronology of the search assets. The searches were not re-run to update the corpus; database-specific yields and recall are therefore not reported.

S1.2. Conceptual blocks and query logic

Block	Content
A — epigenetic ageing	epigenetic age/aging/ageing; DNA methylation age; DNAm age; DNA methylation clock(s); epigenetic clock(s); epigenetic age/aging/ageing acceleration; age acceleration residual; Horvath; Hannum; PhenoAge; GrimAge; DunedinPACE; DunedinPoAm
BA — biological age (not necessarily epigenetic)	biological age; biological aging/ageing; aging/ageing biomarker; biomarker of aging/ageing; molecular age; geroscience biomarker
S — socioeconomic position and social adversity	socioeconomic status/position; education; income; wealth; poverty; deprivation; occupation and social class; social/socioeconomic gradient; social determinants of health; neighbourhood disadvantage; health disparities/inequalities/inequities; economic adversity; economic injustice; social adversity; racialized economic injustice; financial hardship
I — formal inequality measures	Gini coefficient/index; (generalized) concentration index; slope and relative index of inequality; Theil and Atkinson indices; ridit;

Block	Content
	inequality/disparity/distributional/between-group measures; health equity metrics and inequality monitoring
H — reliability and harmonisation	reliability; reproducibility; validation; calibration; harmonization; comparability; transportability; generalizability; measurement invariance; standardization; cross-population/cross-cohort/multicohort; ancestry; race; ethnicity; tissue; platform; cell composition; batch
K — indicators, policy and Agenda 2030	SDG/Agenda 2030; population/public health indicator; health equity indicator; biological equity; biological KPI; health inequality/equity monitoring; population health surveillance; geroscience policy; precision public health

Table S1.2. Conceptual blocks. Gini-type terms were used as bibliographic comparators only.

Query	Question	Boolean logic
Q1	Which associations between socioeconomic position and EAA are already documented?	A AND S
Q2A	Is a formal inequality measure already applied to DNAm-based EAA across socioeconomic strata?	A AND I AND S
Q2B	Is a formal inequality measure applied to non-epigenetic biological age?	BA AND I AND S
Q3	Which reliability and comparability limits affect DNAm clocks?	A AND H
Q4	Are ageing biomarkers proposed as equity, surveillance or SDG indicators?	(A OR BA) AND K

Table S1.3. Operational query families. Q2A and Q2B address the same conceptual question (Q2) and were kept separate so that a non-epigenetic result could not be mistaken for a direct precedent.

S1.3. Platform-specific syntax

- PubMed: single terms searched in [tiab]; multi-word phrases in [Title/Abstract:~0] (zero-distance proximity); publication date limited to 1900/01/01–2026/07/30 [dp].
- Scopus: TITLE-ABS-KEY(…) AND PUBYEAR < 2027; records dated after 30 July 2026 to be removed manually.
- Web of Science Core Collection: TS=(…) AND PY=(1900-2026); records after the cutoff to be removed manually.
- Europe PMC (preprints): every term searched explicitly in TITLE: or ABSTRACT:, with SRC:PPR and FIRST_PDATE:[1900-01-01 TO 2026-07-30].
- Exact-name check: "epigenetic coefficient"[Title/Abstract:~0] (PubMed) and TITLE:"Epigenetic Coefficient" OR ABSTRACT:"Epigenetic Coefficient" (Europe PMC).

S1.4. Full search strings

The 20 strings are reproduced verbatim from the archived UTF-8 files. Characters and Boolean-operator counts refer to the archived file; the SHA-256 value is the hash of the UTF-8 file.

String 1. Q1 — PubMed

Characters: 2067; Boolean operators: 54; SHA-256: 1b0c8a7265352bbd8ce18026eee8809a335e8c42091a86f8227177bbcc29b769

```
(("epigenetic age"[Title/Abstract:~0] OR "epigenetic aging"[Title/Abstract:~0] OR "epigenetic
ageing"[Title/Abstract:~0] OR "DNA methylation age"[Title/Abstract:~0] OR "DNAm age"[Title/Abstract:~0]
OR "DNA methylation clock"[Title/Abstract:~0] OR "DNA methylation clocks"[Title/Abstract:~0] OR
"epigenetic clock"[Title/Abstract:~0] OR "epigenetic clocks"[Title/Abstract:~0] OR "epigenetic age
acceleration"[Title/Abstract:~0] OR "epigenetic aging acceleration"[Title/Abstract:~0] OR "epigenetic
ageing acceleration"[Title/Abstract:~0] OR "age acceleration residual"[Title/Abstract:~0] OR
Horvath[tiab] OR Hannum[tiab] OR PhenoAge[tiab] OR GrimAge[tiab] OR DunedinPACE[tiab] OR
```

```
DunedinPoAm[tiab]) AND (socioeconomic[tiab] OR "socio-economic"[Title/Abstract:~0] OR SES[tiab] OR
"socioeconomic status"[Title/Abstract:~0] OR "socioeconomic position"[Title/Abstract:~0] OR
education[tiab] OR "educational attainment"[Title/Abstract:~0] OR income[tiab] OR wealth[tiab] OR
poverty[tiab] OR deprivation[tiab] OR occupation[tiab] OR "occupational class"[Title/Abstract:~0] OR
employment[tiab] OR "social class"[Title/Abstract:~0] OR "social gradient"[Title/Abstract:~0] OR
"socioeconomic gradient"[Title/Abstract:~0] OR "social determinant of health"[Title/Abstract:~0] OR
"social determinants of health"[Title/Abstract:~0] OR "neighborhood disadvantage"[Title/Abstract:~0] OR
"neighbourhood disadvantage"[Title/Abstract:~0] OR "neighborhood poverty"[Title/Abstract:~0] OR
"neighbourhood poverty"[Title/Abstract:~0] OR "area deprivation"[Title/Abstract:~0] OR "health
disparity"[Title/Abstract:~0] OR "health disparities"[Title/Abstract:~0] OR "health
inequality"[Title/Abstract:~0] OR "health inequalities"[Title/Abstract:~0] OR "health
inequity"[Title/Abstract:~0] OR "health inequities"[Title/Abstract:~0] OR "economic
adversity"[Title/Abstract:~0] OR "economic injustice"[Title/Abstract:~0] OR "social
adversity"[Title/Abstract:~0] OR "racialized economic injustice"[Title/Abstract:~0] OR "financial
hardship"[Title/Abstract:~0])) AND ("1900/01/01"[dp] : "2026/07/30"[dp])
```

String 2. Q1 — Scopus

Characters: 1226; Boolean operators: 54; SHA-256: 767b61deaf35572e5546e5bde39a549e509cf95782838c9c876134a15078e3e7

```
TITLE-ABS-KEY(("epigenetic age" OR "epigenetic aging" OR "epigenetic ageing" OR "DNA methylation age" OR
"DNAm age" OR "DNA methylation clock" OR "DNA methylation clocks" OR "epigenetic clock" OR "epigenetic
clocks" OR "epigenetic age acceleration" OR "epigenetic aging acceleration" OR "epigenetic ageing
acceleration" OR "age acceleration residual" OR Horvath OR Hannum OR PhenoAge OR GrimAge OR DunedinPACE
OR DunedinPoAm) AND (socioeconomic OR "socio-economic" OR SES OR "socioeconomic status" OR
"socioeconomic position" OR education OR "educational attainment" OR income OR wealth OR poverty OR
deprivation OR occupation OR "occupational class" OR employment OR "social class" OR "social gradient"
OR "socioeconomic gradient" OR "social determinant of health" OR "social determinants of health" OR
"neighborhood disadvantage" OR "neighbourhood disadvantage" OR "neighborhood poverty" OR "neighbourhood
poverty" OR "area deprivation" OR "health disparity" OR "health disparities" OR "health inequality" OR
"health inequalities" OR "health inequity" OR "health inequities" OR "economic adversity" OR "economic
injustice" OR "social adversity" OR "racialized economic injustice" OR "financial hardship")) AND
PUBYEAR < 2027
```

String 3. Q1 — Web of Science

Characters: 1216; Boolean operators: 54; SHA-256: e78acf1d69d81ba8495ce9c4938494dd46082b6ab320da867e4e3b4f4a6e7a89

```
TS=(("epigenetic age" OR "epigenetic aging" OR "epigenetic ageing" OR "DNA methylation age" OR "DNAm
age" OR "DNA methylation clock" OR "DNA methylation clocks" OR "epigenetic clock" OR "epigenetic clocks"
OR "epigenetic age acceleration" OR "epigenetic aging acceleration" OR "epigenetic ageing acceleration"
OR "age acceleration residual" OR Horvath OR Hannum OR PhenoAge OR GrimAge OR DunedinPACE OR
DunedinPoAm) AND (socioeconomic OR "socio-economic" OR SES OR "socioeconomic status" OR "socioeconomic
position" OR education OR "educational attainment" OR income OR wealth OR poverty OR deprivation OR
occupation OR "occupational class" OR employment OR "social class" OR "social gradient" OR
"socioeconomic gradient" OR "social determinant of health" OR "social determinants of health" OR
"neighborhood disadvantage" OR "neighbourhood disadvantage" OR "neighborhood poverty" OR "neighbourhood
poverty" OR "area deprivation" OR "health disparity" OR "health disparities" OR "health inequality" OR
"health inequalities" OR "health inequity" OR "health inequities" OR "economic adversity" OR "economic
injustice" OR "social adversity" OR "racialized economic injustice" OR "financial hardship")) AND
PY=(1900-2026)
```

String 4. Q1 — Europe PMC

Characters: 3250; Boolean operators: 109; SHA-256: be12b6da8d0d9bc53a048b36fd86d76fa1f37e36f5ceb59137ed4f80331d6a70

```
((TITLE:"epigenetic age" OR ABSTRACT:"epigenetic age" OR TITLE:"epigenetic aging" OR
ABSTRACT:"epigenetic aging" OR TITLE:"epigenetic ageing" OR ABSTRACT:"epigenetic ageing" OR TITLE:"DNA
methylation age" OR ABSTRACT:"DNA methylation age" OR TITLE:"DNAm age" OR ABSTRACT:"DNAm age" OR
TITLE:"DNA methylation clock" OR ABSTRACT:"DNA methylation clock" OR TITLE:"DNA methylation clocks" OR
ABSTRACT:"DNA methylation clocks" OR TITLE:"epigenetic clock" OR ABSTRACT:"epigenetic clock" OR
TITLE:"epigenetic clocks" OR ABSTRACT:"epigenetic clocks" OR TITLE:"epigenetic age acceleration" OR
ABSTRACT:"epigenetic age acceleration" OR TITLE:"epigenetic aging acceleration" OR ABSTRACT:"epigenetic
aging acceleration" OR TITLE:"epigenetic ageing acceleration" OR ABSTRACT:"epigenetic ageing
acceleration" OR TITLE:"age acceleration residual" OR ABSTRACT:"age acceleration residual" OR
TITLE:Horvath OR ABSTRACT:Horvath OR TITLE:Hannum OR ABSTRACT:Hannum OR TITLE:PhenoAge OR
ABSTRACT:PhenoAge OR TITLE:GrimAge OR ABSTRACT:GrimAge OR TITLE:DunedinPACE OR ABSTRACT:DunedinPACE OR
TITLE:DunedinPoAm OR ABSTRACT:DunedinPoAm) AND (TITLE:socioeconomic OR ABSTRACT:socioeconomic OR
TITLE:"socio-economic" OR ABSTRACT:"socio-economic" OR TITLE:SES OR ABSTRACT:SES OR TITLE:"socioeconomic
status" OR ABSTRACT:"socioeconomic status" OR TITLE:"socioeconomic position" OR ABSTRACT:"socioeconomic
position" OR TITLE:education OR ABSTRACT:education OR TITLE:"educational attainment" OR
ABSTRACT:"educational attainment" OR TITLE:income OR ABSTRACT:income OR TITLE:wealth OR ABSTRACT:wealth
OR TITLE:poverty OR ABSTRACT:poverty OR TITLE:deprivation OR ABSTRACT:deprivation OR TITLE:occupation OR
ABSTRACT:occupation OR TITLE:"occupational class" OR ABSTRACT:"occupational class" OR TITLE:employment
OR ABSTRACT:employment OR TITLE:"social class" OR ABSTRACT:"social class" OR TITLE:"social gradient" OR
ABSTRACT:"social gradient" OR TITLE:"socioeconomic gradient" OR ABSTRACT:"socioeconomic gradient" OR
TITLE:"social determinant of health" OR ABSTRACT:"social determinant of health" OR TITLE:"social
determinants of health" OR ABSTRACT:"social determinants of health" OR TITLE:"neighborhood disadvantage"
OR ABSTRACT:"neighborhood disadvantage" OR TITLE:"neighbourhood disadvantage" OR ABSTRACT:"neighbourhood
```

```
disadvantage" OR TITLE:"neighborhood poverty" OR ABSTRACT:"neighborhood poverty" OR TITLE:"neighbourhood
poverty" OR ABSTRACT:"neighbourhood poverty" OR TITLE:"area deprivation" OR ABSTRACT:"area deprivation"
OR TITLE:"health disparity" OR ABSTRACT:"health disparity" OR TITLE:"health disparities" OR
ABSTRACT:"health disparities" OR TITLE:"health inequality" OR ABSTRACT:"health inequality" OR
TITLE:"health inequalities" OR ABSTRACT:"health inequalities" OR TITLE:"health inequity" OR
ABSTRACT:"health inequity" OR TITLE:"health inequities" OR ABSTRACT:"health inequities" OR
TITLE:"economic adversity" OR ABSTRACT:"economic adversity" OR TITLE:"economic injustice" OR
ABSTRACT:"economic injustice" OR TITLE:"social adversity" OR ABSTRACT:"social adversity" OR
TITLE:"racialized economic injustice" OR ABSTRACT:"racialized economic injustice" OR TITLE:"financial
hardship" OR ABSTRACT:"financial hardship")) AND SRC:PPR AND FIRST_PDATE:[1900-01-01 TO 2026-07-30]
```

String 5. Q2A — PubMed

Characters: 2851; Boolean operators: 72; SHA-256: d40f6910048a0225819f2574a550e3201ebc1de526707a5791512d0dec0e037a

```
(("epigenetic age"[Title/Abstract:~0] OR "epigenetic aging"[Title/Abstract:~0] OR "epigenetic
ageing"[Title/Abstract:~0] OR "DNA methylation age"[Title/Abstract:~0] OR "DNAm age"[Title/Abstract:~0]
OR "DNA methylation clock"[Title/Abstract:~0] OR "DNA methylation clocks"[Title/Abstract:~0] OR
"epigenetic clock"[Title/Abstract:~0] OR "epigenetic clocks"[Title/Abstract:~0] OR "epigenetic age
acceleration"[Title/Abstract:~0] OR "epigenetic aging acceleration"[Title/Abstract:~0] OR "epigenetic
ageing acceleration"[Title/Abstract:~0] OR "age acceleration residual"[Title/Abstract:~0] OR
Horvath[tiab] OR Hannum[tiab] OR PhenoAge[tiab] OR GrimAge[tiab] OR DunedinPACE[tiab] OR
DunedinPoAm[tiab]) AND ("Gini coefficient"[Title/Abstract:~0] OR "Gini index"[Title/Abstract:~0] OR
"concentration index"[Title/Abstract:~0] OR "generalized concentration index"[Title/Abstract:~0] OR
"slope index of inequality"[Title/Abstract:~0] OR "relative index of inequality"[Title/Abstract:~0] OR
"Theil index"[Title/Abstract:~0] OR "Atkinson index"[Title/Abstract:~0] OR ridit[tiab] OR "inequality
index"[Title/Abstract:~0] OR "inequality measure"[Title/Abstract:~0] OR "inequality
metric"[Title/Abstract:~0] OR "disparity index"[Title/Abstract:~0] OR "distributional
inequality"[Title/Abstract:~0] OR "between-group inequality"[Title/Abstract:~0] OR "between group
inequality"[Title/Abstract:~0] OR "health equity metric"[Title/Abstract:~0] OR "health inequality
monitoring"[Title/Abstract:~0]) AND (socioeconomic[tiab] OR "socio-economic"[Title/Abstract:~0] OR
SES[tiab] OR "socioeconomic status"[Title/Abstract:~0] OR "socioeconomic position"[Title/Abstract:~0] OR
education[tiab] OR "educational attainment"[Title/Abstract:~0] OR income[tiab] OR wealth[tiab] OR
poverty[tiab] OR deprivation[tiab] OR occupation[tiab] OR "occupational class"[Title/Abstract:~0] OR
employment[tiab] OR "social class"[Title/Abstract:~0] OR "social gradient"[Title/Abstract:~0] OR
"socioeconomic gradient"[Title/Abstract:~0] OR "social determinant of health"[Title/Abstract:~0] OR
"social determinants of health"[Title/Abstract:~0] OR "neighborhood disadvantage"[Title/Abstract:~0] OR
"neighbourhood disadvantage"[Title/Abstract:~0] OR "neighborhood poverty"[Title/Abstract:~0] OR
"neighbourhood poverty"[Title/Abstract:~0] OR "area deprivation"[Title/Abstract:~0] OR "health
disparity"[Title/Abstract:~0] OR "health disparities"[Title/Abstract:~0] OR "health
inequality"[Title/Abstract:~0] OR "health inequalities"[Title/Abstract:~0] OR "health
inequity"[Title/Abstract:~0] OR "health inequities"[Title/Abstract:~0] OR "economic
adversity"[Title/Abstract:~0] OR "economic injustice"[Title/Abstract:~0] OR "social
adversity"[Title/Abstract:~0] OR "racialized economic injustice"[Title/Abstract:~0] OR "financial
hardship"[Title/Abstract:~0])) AND ("1900/01/01"[dp] : "2026/07/30"[dp])
```

String 6. Q2A — Scopus

Characters: 1681; Boolean operators: 72; SHA-256: 44e1a3d5e2937e73245560fbc54e5c17e57e9738bd05fc5831f1e61b841302cc

```
TITLE-ABS-KEY(("epigenetic age" OR "epigenetic aging" OR "epigenetic ageing" OR "DNA methylation age" OR
"DNAm age" OR "DNA methylation clock" OR "DNA methylation clocks" OR "epigenetic clock" OR "epigenetic
clocks" OR "epigenetic age acceleration" OR "epigenetic aging acceleration" OR "epigenetic ageing
acceleration" OR "age acceleration residual" OR Horvath OR Hannum OR PhenoAge OR GrimAge OR DunedinPACE
OR DunedinPoAm) AND ("Gini coefficient" OR "Gini index" OR "concentration index" OR "generalized
concentration index" OR "slope index of inequality" OR "relative index of inequality" OR "Theil index"
OR "Atkinson index" OR ridit OR "inequality index" OR "inequality measure" OR "inequality metric" OR
"disparity index" OR "distributional inequality" OR "between-group inequality" OR "between group
inequality" OR "health equity metric" OR "health inequality monitoring") AND (socioeconomic OR "socio-
economic" OR SES OR "socioeconomic status" OR "socioeconomic position" OR education OR "educational
attainment" OR income OR wealth OR poverty OR deprivation OR occupation OR "occupational class" OR
employment OR "social class" OR "social gradient" OR "socioeconomic gradient" OR "social determinant of
health" OR "social determinants of health" OR "neighborhood disadvantage" OR "neighbourhood
disadvantage" OR "neighborhood poverty" OR "neighbourhood poverty" OR "area deprivation" OR "health
disparity" OR "health disparities" OR "health inequality" OR "health inequalities" OR "health inequity"
OR "health inequities" OR "economic adversity" OR "economic injustice" OR "social adversity" OR
"racialized economic injustice" OR "financial hardship")) AND PUBYEAR < 2027
```

String 7. Q2A — Web of Science

Characters: 1671; Boolean operators: 72; SHA-256: 946f30b51c8d66d2c75ec53ae15c27f56fd2fa5326d7be34789036803f1fa768

```
TS=(("epigenetic age" OR "epigenetic aging" OR "epigenetic ageing" OR "DNA methylation age" OR "DNAm
age" OR "DNA methylation clock" OR "DNA methylation clocks" OR "epigenetic clock" OR "epigenetic clocks"
OR "epigenetic age acceleration" OR "epigenetic aging acceleration" OR "epigenetic ageing acceleration"
OR "age acceleration residual" OR Horvath OR Hannum OR PhenoAge OR GrimAge OR DunedinPACE OR
DunedinPoAm) AND ("Gini coefficient" OR "Gini index" OR "concentration index" OR "generalized
concentration index" OR "slope index of inequality" OR "relative index of inequality" OR "Theil index"
OR "Atkinson index" OR ridit OR "inequality index" OR "inequality measure" OR "inequality metric" OR
```

```
"disparity index" OR "distributional inequality" OR "between-group inequality" OR "between group
inequality" OR "health equity metric" OR "health inequality monitoring") AND (socioeconomic OR "socio-
economic" OR SES OR "socioeconomic status" OR "socioeconomic position" OR education OR "educational
attainment" OR income OR wealth OR poverty OR deprivation OR occupation OR "occupational class" OR
employment OR "social class" OR "social gradient" OR "socioeconomic gradient" OR "social determinant of
health" OR "social determinants of health" OR "neighborhood disadvantage" OR "neighbourhood
disadvantage" OR "neighborhood poverty" OR "neighbourhood poverty" OR "area deprivation" OR "health
disparity" OR "health disparities" OR "health inequality" OR "health inequalities" OR "health inequity"
OR "health inequities" OR "economic adversity" OR "economic injustice" OR "social adversity" OR
"racialized economic injustice" OR "financial hardship")) AND PY=(1900-2026)
```

String 8. Q2A — Europe PMC

Characters: 4427; Boolean operators: 145; SHA-256: aa32bf3db6f78c5609da3cb53d7af428ec4f761e2ae71994ce5988a8ffe3cd06

```
((TITLE:"epigenetic age" OR ABSTRACT:"epigenetic age" OR TITLE:"epigenetic aging" OR
ABSTRACT:"epigenetic aging" OR TITLE:"epigenetic ageing" OR ABSTRACT:"epigenetic ageing" OR TITLE:"DNA
methylation age" OR ABSTRACT:"DNA methylation age" OR TITLE:"DNAm age" OR ABSTRACT:"DNAm age" OR
TITLE:"DNA methylation clock" OR ABSTRACT:"DNA methylation clock" OR TITLE:"DNA methylation clocks" OR
ABSTRACT:"DNA methylation clocks" OR TITLE:"epigenetic clock" OR ABSTRACT:"epigenetic clock" OR
TITLE:"epigenetic clocks" OR ABSTRACT:"epigenetic clocks" OR TITLE:"epigenetic age acceleration" OR
ABSTRACT:"epigenetic age acceleration" OR TITLE:"epigenetic aging acceleration" OR ABSTRACT:"epigenetic
aging acceleration" OR TITLE:"epigenetic ageing acceleration" OR ABSTRACT:"epigenetic ageing
acceleration" OR TITLE:"age acceleration residual" OR ABSTRACT:"age acceleration residual" OR
TITLE:Horvath OR ABSTRACT:Horvath OR TITLE:Hannum OR ABSTRACT:Hannum OR TITLE:PhenoAge OR
ABSTRACT:PhenoAge OR TITLE:GrimAge OR ABSTRACT:GrimAge OR TITLE:DunedinPACE OR ABSTRACT:DunedinPACE OR
TITLE:DunedinPoAm OR ABSTRACT:DunedinPoAm) AND (TITLE:"Gini coefficient" OR ABSTRACT:"Gini coefficient"
OR TITLE:"Gini index" OR ABSTRACT:"Gini index" OR TITLE:"concentration index" OR ABSTRACT:"concentration
index" OR TITLE:"generalized concentration index" OR ABSTRACT:"generalized concentration index" OR
TITLE:"slope index of inequality" OR ABSTRACT:"slope index of inequality" OR TITLE:"relative index of
inequality" OR ABSTRACT:"relative index of inequality" OR TITLE:"Theil index" OR ABSTRACT:"Theil index"
OR TITLE:"Atkinson index" OR ABSTRACT:"Atkinson index" OR TITLE:ridit OR ABSTRACT:ridit OR
TITLE:"inequality index" OR ABSTRACT:"inequality index" OR TITLE:"inequality measure" OR
ABSTRACT:"inequality measure" OR TITLE:"inequality metric" OR ABSTRACT:"inequality metric" OR
TITLE:"disparity index" OR ABSTRACT:"disparity index" OR TITLE:"distributional inequality" OR
ABSTRACT:"distributional inequality" OR TITLE:"between-group inequality" OR ABSTRACT:"between-group
inequality" OR TITLE:"between group inequality" OR ABSTRACT:"between group inequality" OR TITLE:"health
equity metric" OR ABSTRACT:"health equity metric" OR TITLE:"health inequality monitoring" OR
ABSTRACT:"health inequality monitoring") AND (TITLE:socioeconomic OR ABSTRACT:socioeconomic OR
TITLE:"socio-economic" OR ABSTRACT:"socio-economic" OR TITLE:SES OR ABSTRACT:SES OR TITLE:"socioeconomic
status" OR ABSTRACT:"socioeconomic status" OR TITLE:"socioeconomic position" OR ABSTRACT:"socioeconomic
position" OR TITLE:education OR ABSTRACT:education OR TITLE:"educational attainment" OR
ABSTRACT:"educational attainment" OR TITLE:income OR ABSTRACT:income OR TITLE:wealth OR ABSTRACT:wealth
OR TITLE:poverty OR ABSTRACT:poverty OR TITLE:deprivation OR ABSTRACT:deprivation OR TITLE:occupation OR
ABSTRACT:occupation OR TITLE:"occupational class" OR ABSTRACT:"occupational class" OR TITLE:employment
OR ABSTRACT:employment OR TITLE:"social class" OR ABSTRACT:"social class" OR TITLE:"social gradient" OR
ABSTRACT:"social gradient" OR TITLE:"socioeconomic gradient" OR ABSTRACT:"socioeconomic gradient" OR
TITLE:"social determinant of health" OR ABSTRACT:"social determinant of health" OR TITLE:"social
determinants of health" OR ABSTRACT:"social determinants of health" OR TITLE:"neighborhood disadvantage"
OR ABSTRACT:"neighborhood disadvantage" OR TITLE:"neighbourhood disadvantage" OR ABSTRACT:"neighbourhood
disadvantage" OR TITLE:"neighborhood poverty" OR ABSTRACT:"neighborhood poverty" OR TITLE:"neighbourhood
poverty" OR ABSTRACT:"neighbourhood poverty" OR TITLE:"area deprivation" OR ABSTRACT:"area deprivation"
OR TITLE:"health disparity" OR ABSTRACT:"health disparity" OR TITLE:"health disparities" OR
ABSTRACT:"health disparities" OR TITLE:"health inequality" OR ABSTRACT:"health inequality" OR
TITLE:"health inequalities" OR ABSTRACT:"health inequalities" OR TITLE:"health inequity" OR
ABSTRACT:"health inequity" OR TITLE:"health inequities" OR ABSTRACT:"health inequities" OR
TITLE:"economic adversity" OR ABSTRACT:"economic adversity" OR TITLE:"economic injustice" OR
ABSTRACT:"economic injustice" OR TITLE:"social adversity" OR ABSTRACT:"social adversity" OR
TITLE:"racialized economic injustice" OR ABSTRACT:"racialized economic injustice" OR TITLE:"financial
hardship" OR ABSTRACT:"financial hardship")) AND SRC:PPR AND FIRST_PDATE:[1900-01-01 TO 2026-07-30]
```

String 9. Q2B — PubMed

Characters: 2529; Boolean operators: 62; SHA-256: 16cc27a8b11020d29b6416a888ad732130e9e5a4f379f70f045d841fbe2ccebf

```
(("biological age"[Title/Abstract:~0] OR "biological aging"[Title/Abstract:~0] OR "biological
ageing"[Title/Abstract:~0] OR "aging biomarker"[Title/Abstract:~0] OR "ageing
biomarker"[Title/Abstract:~0] OR "biomarker of aging"[Title/Abstract:~0] OR "biomarker of
ageing"[Title/Abstract:~0] OR "molecular age"[Title/Abstract:~0] OR "geroscience
biomarker"[Title/Abstract:~0]) AND ("Gini coefficient"[Title/Abstract:~0] OR "Gini
index"[Title/Abstract:~0] OR "concentration index"[Title/Abstract:~0] OR "generalized concentration
index"[Title/Abstract:~0] OR "slope index of inequality"[Title/Abstract:~0] OR "relative index of
inequality"[Title/Abstract:~0] OR "Theil index"[Title/Abstract:~0] OR "Atkinson
index"[Title/Abstract:~0] OR ridit[tiab] OR "inequality index"[Title/Abstract:~0] OR "inequality
measure"[Title/Abstract:~0] OR "inequality metric"[Title/Abstract:~0] OR "disparity
index"[Title/Abstract:~0] OR "distributional inequality"[Title/Abstract:~0] OR "between-group
inequality"[Title/Abstract:~0] OR "between group inequality"[Title/Abstract:~0] OR "health equity
metric"[Title/Abstract:~0] OR "health inequality monitoring"[Title/Abstract:~0]) AND
(socioeconomic[tiab] OR "socio-economic"[Title/Abstract:~0] OR SES[tiab] OR "socioeconomic
```

```
status"[Title/Abstract:~0] OR "socioeconomic position"[Title/Abstract:~0] OR education[tiab] OR
"educational attainment"[Title/Abstract:~0] OR income[tiab] OR wealth[tiab] OR poverty[tiab] OR
deprivation[tiab] OR occupation[tiab] OR "occupational class"[Title/Abstract:~0] OR employment[tiab] OR
"social class"[Title/Abstract:~0] OR "social gradient"[Title/Abstract:~0] OR "socioeconomic
gradient"[Title/Abstract:~0] OR "social determinant of health"[Title/Abstract:~0] OR "social
determinants of health"[Title/Abstract:~0] OR "neighborhood disadvantage"[Title/Abstract:~0] OR
"neighbourhood disadvantage"[Title/Abstract:~0] OR "neighborhood poverty"[Title/Abstract:~0] OR
"neighbourhood poverty"[Title/Abstract:~0] OR "area deprivation"[Title/Abstract:~0] OR "health
disparity"[Title/Abstract:~0] OR "health disparities"[Title/Abstract:~0] OR "health
inequality"[Title/Abstract:~0] OR "health inequalities"[Title/Abstract:~0] OR "health
inequity"[Title/Abstract:~0] OR "health inequities"[Title/Abstract:~0] OR "economic
adversity"[Title/Abstract:~0] OR "economic injustice"[Title/Abstract:~0] OR "social
adversity"[Title/Abstract:~0] OR "racialized economic injustice"[Title/Abstract:~0] OR "financial
hardship"[Title/Abstract:~0])) AND ("1900/01/01"[dp] : "2026/07/30"[dp])
```

String 10. Q2B — Scopus

Characters: 1471; Boolean operators: 62; SHA-256: 5cc27834e7c3c4632fef7b88f47943f3acdd71bbf293a9c8974465b2f75fe1c8

```
TITLE-ABS-KEY(("biological age" OR "biological aging" OR "biological ageing" OR "aging biomarker" OR
"ageing biomarker" OR "biomarker of aging" OR "biomarker of ageing" OR "molecular age" OR "geroscience
biomarker") AND ("Gini coefficient" OR "Gini index" OR "concentration index" OR "generalized
concentration index" OR "slope index of inequality" OR "relative index of inequality" OR "Theil index"
OR "Atkinson index" OR ridit OR "inequality index" OR "inequality measure" OR "inequality metric" OR
"disparity index" OR "distributional inequality" OR "between-group inequality" OR "between group
inequality" OR "health equity metric" OR "health inequality monitoring") AND (socioeconomic OR "socio-
economic" OR SES OR "socioeconomic status" OR "socioeconomic position" OR education OR "educational
attainment" OR income OR wealth OR poverty OR deprivation OR occupation OR "occupational class" OR
employment OR "social class" OR "social gradient" OR "socioeconomic gradient" OR "social determinant of
health" OR "social determinants of health" OR "neighborhood disadvantage" OR "neighbourhood
disadvantage" OR "neighborhood poverty" OR "neighbourhood poverty" OR "area deprivation" OR "health
disparity" OR "health disparities" OR "health inequality" OR "health inequalities" OR "health inequity"
OR "health inequities" OR "economic adversity" OR "economic injustice" OR "social adversity" OR
"racialized economic injustice" OR "financial hardship")) AND PUBYEAR < 2027
```

String 11. Q2B — Web of Science

Characters: 1461; Boolean operators: 62; SHA-256: 2d35d1894542f817304286a9236618840e1f23f57db993f18bd0ad201d4ac680

```
TS=(("biological age" OR "biological aging" OR "biological ageing" OR "aging biomarker" OR "ageing
biomarker" OR "biomarker of aging" OR "biomarker of ageing" OR "molecular age" OR "geroscience
biomarker") AND ("Gini coefficient" OR "Gini index" OR "concentration index" OR "generalized
concentration index" OR "slope index of inequality" OR "relative index of inequality" OR "Theil index"
OR "Atkinson index" OR ridit OR "inequality index" OR "inequality measure" OR "inequality metric" OR
"disparity index" OR "distributional inequality" OR "between-group inequality" OR "between group
inequality" OR "health equity metric" OR "health inequality monitoring") AND (socioeconomic OR "socio-
economic" OR SES OR "socioeconomic status" OR "socioeconomic position" OR education OR "educational
attainment" OR income OR wealth OR poverty OR deprivation OR occupation OR "occupational class" OR
employment OR "social class" OR "social gradient" OR "socioeconomic gradient" OR "social determinant of
health" OR "social determinants of health" OR "neighborhood disadvantage" OR "neighbourhood
disadvantage" OR "neighborhood poverty" OR "neighbourhood poverty" OR "area deprivation" OR "health
disparity" OR "health disparities" OR "health inequality" OR "health inequalities" OR "health inequity"
OR "health inequities" OR "economic adversity" OR "economic injustice" OR "social adversity" OR
"racialized economic injustice" OR "financial hardship")) AND PY=(1900-2026)
```

String 12. Q2B — Europe PMC

Characters: 3857; Boolean operators: 125; SHA-256: 5eacca745839026c2b81ec358a693c3a44e8387301b6e59d17aefcd522ffccac

```
((TITLE:"biological age" OR ABSTRACT:"biological age" OR TITLE:"biological aging" OR
ABSTRACT:"biological aging" OR TITLE:"biological ageing" OR ABSTRACT:"biological ageing" OR TITLE:"aging
biomarker" OR ABSTRACT:"aging biomarker" OR TITLE:"ageing biomarker" OR ABSTRACT:"ageing biomarker" OR
TITLE:"biomarker of aging" OR ABSTRACT:"biomarker of aging" OR TITLE:"biomarker of ageing" OR
ABSTRACT:"biomarker of ageing" OR TITLE:"molecular age" OR ABSTRACT:"molecular age" OR
TITLE:"geroscience biomarker" OR ABSTRACT:"geroscience biomarker") AND (TITLE:"Gini coefficient" OR
ABSTRACT:"Gini coefficient" OR TITLE:"Gini index" OR ABSTRACT:"Gini index" OR TITLE:"concentration
index" OR ABSTRACT:"concentration index" OR TITLE:"generalized concentration index" OR
ABSTRACT:"generalized concentration index" OR TITLE:"slope index of inequality" OR ABSTRACT:"slope index
of inequality" OR TITLE:"relative index of inequality" OR ABSTRACT:"relative index of inequality" OR
TITLE:"Theil index" OR ABSTRACT:"Theil index" OR TITLE:"Atkinson index" OR ABSTRACT:"Atkinson index" OR
TITLE:ridit OR ABSTRACT:ridit OR TITLE:"inequality index" OR ABSTRACT:"inequality index" OR
TITLE:"inequality measure" OR ABSTRACT:"inequality measure" OR TITLE:"inequality metric" OR
ABSTRACT:"inequality metric" OR TITLE:"disparity index" OR ABSTRACT:"disparity index" OR
TITLE:"distributional inequality" OR ABSTRACT:"distributional inequality" OR TITLE:"between-group
inequality" OR ABSTRACT:"between-group inequality" OR TITLE:"between group inequality" OR
ABSTRACT:"between group inequality" OR TITLE:"health equity metric" OR ABSTRACT:"health equity metric"
OR TITLE:"health inequality monitoring" OR ABSTRACT:"health inequality monitoring") AND
(TITLE:socioeconomic OR ABSTRACT:socioeconomic OR TITLE:"socio-economic" OR ABSTRACT:"socio-economic" OR
TITLE:SES OR ABSTRACT:SES OR TITLE:"socioeconomic status" OR ABSTRACT:"socioeconomic status" OR
```

```
TITLE:"socioeconomic position" OR ABSTRACT:"socioeconomic position" OR TITLE:education OR
ABSTRACT:education OR TITLE:"educational attainment" OR ABSTRACT:"educational attainment" OR
TITLE:income OR ABSTRACT:income OR TITLE:wealth OR ABSTRACT:wealth OR TITLE:poverty OR ABSTRACT:poverty
OR TITLE:deprivation OR ABSTRACT:deprivation OR TITLE:occupation OR ABSTRACT:occupation OR
TITLE:"occupational class" OR ABSTRACT:"occupational class" OR TITLE:employment OR ABSTRACT:employment
OR TITLE:"social class" OR ABSTRACT:"social class" OR TITLE:"social gradient" OR ABSTRACT:"social
gradient" OR TITLE:"socioeconomic gradient" OR ABSTRACT:"socioeconomic gradient" OR TITLE:"social
determinant of health" OR ABSTRACT:"social determinant of health" OR TITLE:"social determinants of
health" OR ABSTRACT:"social determinants of health" OR TITLE:"neighborhood disadvantage" OR
ABSTRACT:"neighborhood disadvantage" OR TITLE:"neighbourhood disadvantage" OR ABSTRACT:"neighbourhood
disadvantage" OR TITLE:"neighborhood poverty" OR ABSTRACT:"neighborhood poverty" OR TITLE:"neighbourhood
poverty" OR ABSTRACT:"neighbourhood poverty" OR TITLE:"area deprivation" OR ABSTRACT:"area deprivation"
OR TITLE:"health disparity" OR ABSTRACT:"health disparity" OR TITLE:"health disparities" OR
ABSTRACT:"health disparities" OR TITLE:"health inequality" OR ABSTRACT:"health inequality" OR
TITLE:"health inequalities" OR ABSTRACT:"health inequalities" OR TITLE:"health inequity" OR
ABSTRACT:"health inequity" OR TITLE:"health inequities" OR ABSTRACT:"health inequities" OR
TITLE:"economic adversity" OR ABSTRACT:"economic adversity" OR TITLE:"economic injustice" OR
ABSTRACT:"economic injustice" OR TITLE:"social adversity" OR ABSTRACT:"social adversity" OR
TITLE:"racialized economic injustice" OR ABSTRACT:"racialized economic injustice" OR TITLE:"financial
hardship" OR ABSTRACT:"financial hardship")) AND SRC:PPR AND FIRST_PDATE:[1900-01-01 TO 2026-07-30]
```

String 13. Q3 — PubMed

Characters: 1427; Boolean operators: 45; SHA-256: 4034d482292273e04d09f46478fa3c67321be9bc7b2c80b990c701bbe0260482

```
(("epigenetic age"[Title/Abstract:~0] OR "epigenetic aging"[Title/Abstract:~0] OR "epigenetic
ageing"[Title/Abstract:~0] OR "DNA methylation age"[Title/Abstract:~0] OR "DNAm age"[Title/Abstract:~0]
OR "DNA methylation clock"[Title/Abstract:~0] OR "DNA methylation clocks"[Title/Abstract:~0] OR
"epigenetic clock"[Title/Abstract:~0] OR "epigenetic clocks"[Title/Abstract:~0] OR "epigenetic age
acceleration"[Title/Abstract:~0] OR "epigenetic aging acceleration"[Title/Abstract:~0] OR "epigenetic
ageing acceleration"[Title/Abstract:~0] OR "age acceleration residual"[Title/Abstract:~0] OR
Horvath[tiab] OR Hannum[tiab] OR PhenoAge[tiab] OR GrimAge[tiab] OR DunedinPACE[tiab] OR
DunedinPoAm[tiab]) AND (reliability[tiab] OR reproducibility[tiab] OR validation[tiab] OR
calibration[tiab] OR harmonization[tiab] OR harmonisation[tiab] OR comparability[tiab] OR
transportability[tiab] OR generalizability[tiab] OR generalisability[tiab] OR "measurement
invariance"[Title/Abstract:~0] OR standardization[tiab] OR standardisation[tiab] OR "cross-
population"[Title/Abstract:~0] OR "cross population"[Title/Abstract:~0] OR "cross-
cohort"[Title/Abstract:~0] OR "cross cohort"[Title/Abstract:~0] OR multicohort[tiab] OR "multi-
cohort"[Title/Abstract:~0] OR ancestry[tiab] OR race[tiab] OR ethnicity[tiab] OR tissue[tiab] OR
platform[tiab] OR "cell composition"[Title/Abstract:~0] OR batch[tiab])) AND ("1900/01/01"[dp] :
"2026/07/30"[dp])
```

String 14. Q3 — Scopus

Characters: 887; Boolean operators: 45; SHA-256: 893af83249e489e4bacb8cb65cec52e200b3d2bf746c9ec2d1ea423a99c84663

```
TITLE-ABS-KEY(("epigenetic age" OR "epigenetic aging" OR "epigenetic ageing" OR "DNA methylation age" OR
"DNAm age" OR "DNA methylation clock" OR "DNA methylation clocks" OR "epigenetic clock" OR "epigenetic
clocks" OR "epigenetic age acceleration" OR "epigenetic aging acceleration" OR "epigenetic ageing
acceleration" OR "age acceleration residual" OR Horvath OR Hannum OR PhenoAge OR GrimAge OR DunedinPACE
OR DunedinPoAm) AND (reliability OR reproducibility OR validation OR calibration OR harmonization OR
harmonisation OR comparability OR transportability OR generalizability OR generalisability OR
"measurement invariance" OR standardization OR standardisation OR "cross-population" OR "cross
population" OR "cross-cohort" OR "cross cohort" OR multicohort OR "multi-cohort" OR ancestry OR race OR
ethnicity OR tissue OR platform OR "cell composition" OR batch)) AND PUBYEAR < 2027
```

String 15. Q3 — Web of Science

Characters: 877; Boolean operators: 45; SHA-256: 025ff05547582db6dd032f359c705f088e7a60320c402767d65e0036e2a67d6a

```
TS=(("epigenetic age" OR "epigenetic aging" OR "epigenetic ageing" OR "DNA methylation age" OR "DNAm
age" OR "DNA methylation clock" OR "DNA methylation clocks" OR "epigenetic clock" OR "epigenetic clocks"
OR "epigenetic age acceleration" OR "epigenetic aging acceleration" OR "epigenetic ageing acceleration"
OR "age acceleration residual" OR Horvath OR Hannum OR PhenoAge OR GrimAge OR DunedinPACE OR
DunedinPoAm) AND (reliability OR reproducibility OR validation OR calibration OR harmonization OR
harmonisation OR comparability OR transportability OR generalizability OR generalisability OR
"measurement invariance" OR standardization OR standardisation OR "cross-population" OR "cross
population" OR "cross-cohort" OR "cross cohort" OR multicohort OR "multi-cohort" OR ancestry OR race OR
ethnicity OR tissue OR platform OR "cell composition" OR batch)) AND PY=(1900-2026)
```

String 16. Q3 — Europe PMC

Characters: 2437; Boolean operators: 91; SHA-256: 8b99c3ad02c9484dc6b9a4b467c4b8b918c9e3237378c0436b01da70b1a59b41

```
((TITLE:"epigenetic age" OR ABSTRACT:"epigenetic age" OR TITLE:"epigenetic aging" OR
ABSTRACT:"epigenetic aging" OR TITLE:"epigenetic ageing" OR ABSTRACT:"epigenetic ageing" OR TITLE:"DNA
methylation age" OR ABSTRACT:"DNA methylation age" OR TITLE:"DNAm age" OR ABSTRACT:"DNAm age" OR
TITLE:"DNA methylation clock" OR ABSTRACT:"DNA methylation clock" OR TITLE:"DNA methylation clocks" OR
ABSTRACT:"DNA methylation clocks" OR TITLE:"epigenetic clock" OR ABSTRACT:"epigenetic clock" OR
```

```
TITLE:"epigenetic clocks" OR ABSTRACT:"epigenetic clocks" OR TITLE:"epigenetic age acceleration" OR
ABSTRACT:"epigenetic age acceleration" OR TITLE:"epigenetic aging acceleration" OR ABSTRACT:"epigenetic
aging acceleration" OR TITLE:"epigenetic ageing acceleration" OR ABSTRACT:"epigenetic ageing
acceleration" OR TITLE:"age acceleration residual" OR ABSTRACT:"age acceleration residual" OR
TITLE:Horvath OR ABSTRACT:Horvath OR TITLE:Hannum OR ABSTRACT:Hannum OR TITLE:PhenoAge OR
ABSTRACT:PhenoAge OR TITLE:GrimAge OR ABSTRACT:GrimAge OR TITLE:DunedinPACE OR ABSTRACT:DunedinPACE OR
TITLE:DunedinPoAm OR ABSTRACT:DunedinPoAm) AND (TITLE:reliability OR ABSTRACT:reliability OR
TITLE:reproducibility OR ABSTRACT:reproducibility OR TITLE:validation OR ABSTRACT:validation OR
TITLE:calibration OR ABSTRACT:calibration OR TITLE:harmonization OR ABSTRACT:harmonization OR
TITLE:harmonisation OR ABSTRACT:harmonisation OR TITLE:comparability OR ABSTRACT:comparability OR
TITLE:transportability OR ABSTRACT:transportability OR TITLE:generalizability OR
ABSTRACT:generalizability OR TITLE:generalisability OR ABSTRACT:generalisability OR TITLE:"measurement
invariance" OR ABSTRACT:"measurement invariance" OR TITLE:standardization OR ABSTRACT:standardization OR
TITLE:standardisation OR ABSTRACT:standardisation OR TITLE:"cross-population" OR ABSTRACT:"cross-
population" OR TITLE:"cross population" OR ABSTRACT:"cross population" OR TITLE:"cross-cohort" OR
ABSTRACT:"cross-cohort" OR TITLE:"cross cohort" OR ABSTRACT:"cross cohort" OR TITLE:multicohort OR
ABSTRACT:multicohort OR TITLE:"multi-cohort" OR ABSTRACT:"multi-cohort" OR TITLE:ancestry OR
ABSTRACT:ancestry OR TITLE:race OR ABSTRACT:race OR TITLE:ethnicity OR ABSTRACT:ethnicity OR
TITLE:tissue OR ABSTRACT:tissue OR TITLE:platform OR ABSTRACT:platform OR TITLE:"cell composition" OR
ABSTRACT:"cell composition" OR TITLE:batch OR ABSTRACT:batch)) AND SRC:PPR AND FIRST_PDATE:[1900-01-01
TO 2026-07-30]
```

String 17. Q4 — PubMed

Characters: 1854; Boolean operators: 46; SHA-256: 62234dcba54d6c1000a995f347a0726a7f21d99ba142ebda673e0aaf78de071f

```
(("epigenetic age"[Title/Abstract:~0] OR "epigenetic aging"[Title/Abstract:~0] OR "epigenetic
ageing"[Title/Abstract:~0] OR "DNA methylation age"[Title/Abstract:~0] OR "DNAm age"[Title/Abstract:~0]
OR "DNA methylation clock"[Title/Abstract:~0] OR "DNA methylation clocks"[Title/Abstract:~0] OR
"epigenetic clock"[Title/Abstract:~0] OR "epigenetic clocks"[Title/Abstract:~0] OR "epigenetic age
acceleration"[Title/Abstract:~0] OR "epigenetic aging acceleration"[Title/Abstract:~0] OR "epigenetic
ageing acceleration"[Title/Abstract:~0] OR "age acceleration residual"[Title/Abstract:~0] OR
Horvath[tiab] OR Hannum[tiab] OR PhenoAge[tiab] OR GrimAge[tiab] OR DunedinPACE[tiab] OR
DunedinPoAm[tiab] OR "biological age"[Title/Abstract:~0] OR "biological aging"[Title/Abstract:~0] OR
"biological ageing"[Title/Abstract:~0] OR "aging biomarker"[Title/Abstract:~0] OR "ageing
biomarker"[Title/Abstract:~0] OR "biomarker of aging"[Title/Abstract:~0] OR "biomarker of
ageing"[Title/Abstract:~0] OR "molecular age"[Title/Abstract:~0] OR "geroscience
biomarker"[Title/Abstract:~0]) AND ("Sustainable Development Goal"[Title/Abstract:~0] OR "Sustainable
Development Goals"[Title/Abstract:~0] OR "SDG 3"[Title/Abstract:~0] OR SDG3[tiab] OR "SDG
10"[Title/Abstract:~0] OR SDG10[tiab] OR "Agenda 2030"[Title/Abstract:~0] OR "2030
Agenda"[Title/Abstract:~0] OR "population health indicator"[Title/Abstract:~0] OR "public health
indicator"[Title/Abstract:~0] OR "health equity indicator"[Title/Abstract:~0] OR "biological
equity"[Title/Abstract:~0] OR "biological KPI"[Title/Abstract:~0] OR "health inequality
monitoring"[Title/Abstract:~0] OR "equity monitoring"[Title/Abstract:~0] OR "population health
surveillance"[Title/Abstract:~0] OR "geroscience policy"[Title/Abstract:~0] OR "precision public
health"[Title/Abstract:~0])) AND ("1900/01/01"[dp] : "2026/07/30"[dp])
```

String 18. Q4 — Scopus

Characters: 1074; Boolean operators: 46; SHA-256: e2e692846968e7d4bc8df21651480582108d5b37306b45981798d1908cd2c202

```
TITLE-ABS-KEY(("epigenetic age" OR "epigenetic aging" OR "epigenetic ageing" OR "DNA methylation age" OR
"DNAm age" OR "DNA methylation clock" OR "DNA methylation clocks" OR "epigenetic clock" OR "epigenetic
clocks" OR "epigenetic age acceleration" OR "epigenetic aging acceleration" OR "epigenetic ageing
acceleration" OR "age acceleration residual" OR Horvath OR Hannum OR PhenoAge OR GrimAge OR DunedinPACE
OR DunedinPoAm OR "biological age" OR "biological aging" OR "biological ageing" OR "aging biomarker" OR
"ageing biomarker" OR "biomarker of aging" OR "biomarker of ageing" OR "molecular age" OR "geroscience
biomarker") AND ("Sustainable Development Goal" OR "Sustainable Development Goals" OR "SDG 3" OR SDG3 OR
"SDG 10" OR SDG10 OR "Agenda 2030" OR "2030 Agenda" OR "population health indicator" OR "public health
indicator" OR "health equity indicator" OR "biological equity" OR "biological KPI" OR "health inequality
monitoring" OR "equity monitoring" OR "population health surveillance" OR "geroscience policy" OR
"precision public health")) AND PUBYEAR < 2027
```

String 19. Q4 — Web of Science

Characters: 1064; Boolean operators: 46; SHA-256: 917ecf81564b11912d28175362a885e71b2ed3e1c1e4a7a2dc63389c305f34fa

```
TS=(("epigenetic age" OR "epigenetic aging" OR "epigenetic ageing" OR "DNA methylation age" OR "DNAm
age" OR "DNA methylation clock" OR "DNA methylation clocks" OR "epigenetic clock" OR "epigenetic clocks"
OR "epigenetic age acceleration" OR "epigenetic aging acceleration" OR "epigenetic ageing acceleration"
OR "age acceleration residual" OR Horvath OR Hannum OR PhenoAge OR GrimAge OR DunedinPACE OR DunedinPoAm
OR "biological age" OR "biological aging" OR "biological ageing" OR "aging biomarker" OR "ageing
biomarker" OR "biomarker of aging" OR "biomarker of ageing" OR "molecular age" OR "geroscience
biomarker") AND ("Sustainable Development Goal" OR "Sustainable Development Goals" OR "SDG 3" OR SDG3 OR
"SDG 10" OR SDG10 OR "Agenda 2030" OR "2030 Agenda" OR "population health indicator" OR "public health
indicator" OR "health equity indicator" OR "biological equity" OR "biological KPI" OR "health inequality
monitoring" OR "equity monitoring" OR "population health surveillance" OR "geroscience policy" OR
"precision public health")) AND PY=(1900-2026)
```

String 20. Q4 — Europe PMC

Characters: 2826; Boolean operators: 93; SHA-256: 78e197eca997945f7dbe796a982eb599077a9d596326cb5a8a6fbc74c4f19061

```
((TITLE:"epigenetic age" OR ABSTRACT:"epigenetic age" OR TITLE:"epigenetic aging" OR
ABSTRACT:"epigenetic aging" OR TITLE:"epigenetic ageing" OR ABSTRACT:"epigenetic ageing" OR TITLE:"DNA
methylation age" OR ABSTRACT:"DNA methylation age" OR TITLE:"DNAm age" OR ABSTRACT:"DNAm age" OR
TITLE:"DNA methylation clock" OR ABSTRACT:"DNA methylation clock" OR TITLE:"DNA methylation clocks" OR
ABSTRACT:"DNA methylation clocks" OR TITLE:"epigenetic clock" OR ABSTRACT:"epigenetic clock" OR
TITLE:"epigenetic clocks" OR ABSTRACT:"epigenetic clocks" OR TITLE:"epigenetic age acceleration" OR
ABSTRACT:"epigenetic age acceleration" OR TITLE:"epigenetic aging acceleration" OR ABSTRACT:"epigenetic
aging acceleration" OR TITLE:"epigenetic ageing acceleration" OR ABSTRACT:"epigenetic ageing
acceleration" OR TITLE:"age acceleration residual" OR ABSTRACT:"age acceleration residual" OR
TITLE:Horvath OR ABSTRACT:Horvath OR TITLE:Hannum OR ABSTRACT:Hannum OR TITLE:PhenoAge OR
ABSTRACT:PhenoAge OR TITLE:GrimAge OR ABSTRACT:GrimAge OR TITLE:DunedinPACE OR ABSTRACT:DunedinPACE OR
TITLE:DunedinPoAm OR ABSTRACT:DunedinPoAm OR TITLE:"biological age" OR ABSTRACT:"biological age" OR
TITLE:"biological aging" OR ABSTRACT:"biological aging" OR TITLE:"biological ageing" OR
ABSTRACT:"biological ageing" OR TITLE:"aging biomarker" OR ABSTRACT:"aging biomarker" OR TITLE:"ageing
biomarker" OR ABSTRACT:"ageing biomarker" OR TITLE:"biomarker of aging" OR ABSTRACT:"biomarker of aging"
OR TITLE:"biomarker of ageing" OR ABSTRACT:"biomarker of ageing" OR TITLE:"molecular age" OR
ABSTRACT:"molecular age" OR TITLE:"geroscience biomarker" OR ABSTRACT:"geroscience biomarker") AND
(TITLE:"Sustainable Development Goal" OR ABSTRACT:"Sustainable Development Goal" OR TITLE:"Sustainable
Development Goals" OR ABSTRACT:"Sustainable Development Goals" OR TITLE:"SDG 3" OR ABSTRACT:"SDG 3" OR
TITLE:SDG3 OR ABSTRACT:SDG3 OR TITLE:"SDG 10" OR ABSTRACT:"SDG 10" OR TITLE:SDG10 OR ABSTRACT:SDG10 OR
TITLE:"Agenda 2030" OR ABSTRACT:"Agenda 2030" OR TITLE:"2030 Agenda" OR ABSTRACT:"2030 Agenda" OR
TITLE:"population health indicator" OR ABSTRACT:"population health indicator" OR TITLE:"public health
indicator" OR ABSTRACT:"public health indicator" OR TITLE:"health equity indicator" OR ABSTRACT:"health
equity indicator" OR TITLE:"biological equity" OR ABSTRACT:"biological equity" OR TITLE:"biological KPI"
OR ABSTRACT:"biological KPI" OR TITLE:"health inequality monitoring" OR ABSTRACT:"health inequality
monitoring" OR TITLE:"equity monitoring" OR ABSTRACT:"equity monitoring" OR TITLE:"population health
surveillance" OR ABSTRACT:"population health surveillance" OR TITLE:"geroscience policy" OR
ABSTRACT:"geroscience policy" OR TITLE:"precision public health" OR ABSTRACT:"precision public health"))
AND SRC:PPR AND FIRST_PDATE:[1900-01-01 TO 2026-07-30]
```

S1.5. Deduplication rules

Deduplication distinguished three levels: the physical file (one PDF), the publication (an article and its linked versions) and the canonical record used for screening.

- Automatic merging required at least one of: identical normalized full text; identical resolved DOI passing a frozen syntactic validation rule; or identical normalized title that was non-generic and at least 40 characters long. Incomplete DOI fragments were never used to create links.
- Fuzzy title matches were not merged automatically; they were reviewed manually.
- Preprints and their published versions were linked as one publication; the peer-reviewed version was retained as the canonical record unless data were available only in the preprint.
- Within each cluster the canonical file was chosen deterministically: published version over preprint; valid DOI; informative title; larger amount of text; larger file; file name as final tie-breaker.
- Separate analyses of the same cohort were kept as distinct publications when questions, samples or outcomes differed.
- Folder or file-name hints were not used to infer the original query or platform.

**Relation**	**Records**
Canonical publications	238
Duplicate by identical normalized full text	26
Additional file/version of an already canonical publication	20
Duplicate by resolved DOI	14
Duplicate by exact normalized title	6
Preprint linked to its published version	3
Total physical PDF records	307

Table S1.4. Deduplication of the 307 physical records. Eleven publication-level preprint–published-version links were resolved manually during screening. The resulting non-canonical physical records are included in the row "Additional file/version of an already canonical publication".

S1.6. Eligibility and screening rules

Each canonical publication was screened at title/abstract level and, when retained, at full-text level. Three dimensions were recorded separately for every publication: (i) the screening decision (include or exclude); (ii) the role of the evidence (empirical socioeconomic–EAA study, methodological or contextual source, or mapping review); and (iii) proximity to the EGC (precedent class P0–P4 or not pertinent, Section S1.7). A publication could therefore be relevant to the EGC without being an empirical socioeconomic–EAA study.

Inclusion. A publication was included when it (a) reported an association, gradient, contrast, mediation or decomposition between socioeconomic position and an ageing biomarker, preferably DNAm-based EAA; or (b) described an inequality, distributional or monitoring method, a clock-comparability issue or a policy/SDG use of ageing biomarkers that was directly relevant to the construction or interpretation of the EGC; or (c) was a review usable for evidence mapping.

Exclusion. Excluded publications received a single primary reason, assigned hierarchically. Reviews were retained as mapping sources, while primary studies remained the unit of empirical evidence.

**Primary exclusion reason**	**n**
Clock or disease study without a socioeconomic/inequality exposure	23
Mixed adversity or non-socioeconomic exposure	13
Not DNAm-EAA or otherwise out of scope	9
No autonomous socioeconomic–EAA estimate	8
Socioeconomic position used only as a covariate	7
Environmental exposure without a socioeconomic result	3
No biological-ageing outcome	2
Mixed adversity without an isolated socioeconomic–EAA result	2
Race/structural racism without a direct socioeconomic–EAA estimate	1
Non-socioeconomic social exposure	1
Social exposure without a socioeconomic–EAA result	1
No socioeconomic content and no inequality method	1
Income inequality used as a contextual exposure, not as an outcome distribution	1
Corrigendum (not independent evidence)	1
No DNAm-EAA outcome	1
No socioeconomic–EAA result and no clock validation	1
Inequality indicator used only as input to a composite index	1
Non-health distribution without direct methodological transfer	1
No direct socioeconomic–EAA estimate	1
Total excluded	78

Table S1.5. Primary reasons for exclusion (78 publications: 7 at title/abstract level, 71 at full text).

S1.7. Precedent classes P0–P4 and NP

Every publication was classified for proximity to the EGC using five criteria: (1) human biological ageing; (2) DNAm-based EAA; (3) ordered socioeconomic strata; (4) an explicit distributional inequality estimand;

and (5) a named synthetic coefficient or index developed for the socioeconomic distribution of EAA. P0 requires all five criteria; in the main text, criteria 1 and 2 (human biological ageing and DNAm-EAA) are summarized jointly as “human DNAm-EAA”.

Class	Definition	Implication for the novelty claim
P0 — equivalent	Meets all of: human DNAm-EAA; ordered socioeconomic strata; an explicit distributional inequality estimand; and a named synthetic coefficient or index developed for that purpose.	Would refute or restrict the claim of methodological distinctiveness.
P1 — near-direct precedent	Addresses several, but not all, P0 criteria (e.g., a formal inequality or decomposition method applied to biological age, or EAA across socioeconomic groups with index-like formalization).	Requires explicit comparison; all P1 publications underwent full-text claim-level audit (Table S1, sheet 'P1 audit').
P2 — empirical association	Association, gradient, contrast or mediation between socioeconomic position and EAA, without a new distributional index.	Supports the need for the EGC; does not affect novelty.
P3 — methodological context	Health-inequality or distributional method not applied to EAA, or measurement context.	Comparator and mathematical background.
P4 — broader context	Ageing biomarkers or geroscience linked to equity monitoring, surveillance or SDGs, without an EGC-type coefficient.	Positioning precedent, not a formula precedent.
NP — not pertinent	Outside the conceptual or methodological scope, including lexical false positives.	Excluded.

Table S1.6. Precedent classes. One included publication (EGC-P0014, a multicohort comparison of clocks, telomere length and mortality) was retained as clock-comparability context but could not be assigned to P0–P4 because it addresses none of the inequality criteria; it is flagged in Table S1.

S1.8. Summary of the evidence map

Step	n
Physical PDF records	307
Canonical publications after deduplication	238
Excluded at title/abstract	7
Full texts assessed	231
Excluded at full text	71
Included publications	160
Empirical socioeconomic–EAA studies	106
Methodological or contextual sources	45
Mapping reviews	9
P0 (equivalent coefficient)	0
P1 (near-direct precedent)	8
P2	117
P3	23
P4	11
Included, not assignable to P0–P4	1

Step	n
NP (excluded)	78

Table S1.7. Counts of the structured evidence map. Counts describe the frozen developmental corpus and are not PRISMA yields.
